# Placental Insulin-like Growth Factor Signaling and Neurodevelopment: Emerging Evidence from the Placenta–Brain Axis

**DOI:** 10.3390/cells15191759

**Published:** 2026-09-27

**Authors:** Annemarie J. Carver, Eleanor G. Williamson, Hanna E. Stevens

**Affiliations:** 1 Interdisciplinary Graduate Program in Genetics, University of Iowa, Iowa City, IA 52246, USA; 2 Department of Psychiatry, Carver College of Medicine, University of Iowa, Iowa City, IA 52246, USA; nora-williamson@uiowa.edu; 3 Iowa Neuroscience Institute, Carver College of Medicine, University of Iowa, Iowa City, IA 52246, USA; 4 Department of Pediatrics, Massachusetts General Hospital, Harvard Medical School, Boston, MA 02114, USA; 5 Lurie Center for Autism, Massachusetts General Hospital, Harvard Medical School, Boston, MA 02114, USA; 6 Iowa Discovering Research Experiences and Mentorship Postbaccalaureate Program, University of Iowa, Iowa City, IA 52246, USA; 7 Hawk-Intellectual and Developmental Disabilities Research Center, University of Iowa, Iowa City, IA 52246, USA

**Keywords:** neuroplacentology, neurodevelopment, placenta, insulin-like growth factors, IGF signaling, placenta–brain-axis, inflammation

## Abstract

**Highlights:**

**What are the main findings?**

- Placental insulin-like growth factor signaling is implicated in neurodevelopment.
- Common perinatal conditions associated with abnormal neurodevelopment demonstrate altered placental insulin-like growth factor signaling.

**What are the implications of the main findings?**

- Changes in placental insulin-like growth signaling may adversely affect neurodevelopment relevant to neurodevelopmental disorders.
- Placental insulin-like growth factor signaling may play a role in altered neurodevelopment in inflammatory perinatal conditions.

**Abstract:**

The field of neuroplacentology is a growing area that focuses on the influence of placental function on brain development. This field has expanded our understanding of the placenta’s crucial role in proper neurodevelopment as well as placental anomalies that increase the risk of adverse neurodevelopmental outcomes. Neuroplacentology studies have revealed distinct roles of placentally provided hormones and nutrients in neurodevelopment whose disruption by perinatal problems, including inflammation, can increase the risk of neurodevelopmental disorders such as autism spectrum disorder and ADHD. Placental insulin-like growth factor signaling significantly influences brain growth in animal model studies and is tightly linked with human neurodevelopmental outcomes. Insulin-like growth factor signaling is well-established as a regulator of placental function and is fundamental in fetal brain cell developmental processes and plays a role in inflammation. Placental insulin-like growth factor signaling underlies the placental production and delivery of hormones and nutrients that are necessary for fetal neural cell proliferation, differentiation, and growth. It is especially important to study this pathway in the placenta due to aberrant insulin-like growth factor signaling found in conditions such as preterm birth, fetal growth restriction, gestational diabetes, and obesity. All these conditions are linked to a greater risk for neurodevelopmental disorders, neuroinflammation, and other anomalies. This research subfield has also revealed sex differences in the insulin-like growth factor signaling pathway that will contribute to understanding the etiologies of these conditions. This area of study has and will continue to identify specific mechanisms, many currently in model systems, which could eventually be leveraged in preventative and interventive care to improve neurodevelopmental outcomes.

## 1. Introduction

The influence of placental function on neurodevelopment is a growing field of biology referred to as neuroplacentology or the placenta–brain axis. Placental anomalies such as insufficiency, malperfusion, and abnormal morphology have been associated with adverse neurodevelopmental outcomes [1,2,3,4]. Studies in the field of neuroplacentology have revealed significant influences of specific placental-produced hormones and factors on brain developmental processes. The studies have included factors such as allopregnanolone, serotonin, and placental growth factor (PlGF), all of which significantly alter neurobiology, initially and persistently, and eventually also behavior [5,6,7,8,9]. Some of these studies have shown changes to these factors in the context of placental and maternal inflammation, processes linked with both perinatal adversity and abnormalities in neurodevelopment [10,11,12]. These studies have revealed that placental changes often result in highly sex-specific outcomes in fetal neurodevelopment [6,7,13,14,15,16]. These studies sometimes find that males present with more severe phenotypes than females in these conditions, highlighting increased male susceptibilities. This phenomenon is commonly seen in both placental anomalies and neurodevelopmental disorders, as males are often disproportionally more affected than females [17,18,19,20]. While males often display more adverse effects, this is not always the case, as seen in studies that will be discussed in this review. These studies demonstrate that placental signaling likely influences neurodevelopment differently in sexes in ways highly dependent on developmental timing and context. Despite the known importance of these placental factors, there is still a significant gap in understanding the role of specific placental pathways in how neurodevelopment is directed.

A key pathway of interest in this area has been insulin-like growth factor (IGF) signaling. IGF signaling is well-established to regulate both placental function and neurodevelopment [21,22]. Differences in IGF signaling have been linked to both placental anomalies [23,24,25] and neurodevelopmental disorders (NDDs) [26,27,28,29,30,31,32,33]. Placental IGF signaling differences are associated with common conditions involving dysregulated inflammatory mediators such as preterm birth, fetal growth restriction, obesity, and gestational diabetes, which are linked to increased NDD-risk [25,34,35,36,37,38,39,40,41,42,43]. IGF signaling changes also interact with placental inflammatory processes that are linked to these perinatal conditions and NDD-risk [10,11,12]. There is burgeoning research focusing on the impact of placental IGF signaling on neurodevelopment from human and animal studies. These studies have teased out specific effects of placental factors in the IGF signaling pathway on neurodevelopment, often with a focus on sex-specific outcomes. These studies help characterize the basic biology of how IGF signaling in placenta, a tissue that can have dramatic inflammatory changes with perinatal adversity, plays a role in neurodevelopment. The objective of this review was to synthesize the current literature that demonstrates changes in placental IGF signaling and alterations in neurodevelopment both from human and animal studies. To identify studies that demonstrate both changes to placental IGF signaling and altered neurodevelopment, a structured narrative search was performed between January and July 2026 on PubMed and Google Scholar to identify articles with both components by using keywords including placenta, IGF, insulin-like growth factor, brain, and neurodevelopment. Citation lists of the identified papers were also utilized to identify any additional studies. It is critical to understand the influence of these pathways on neurodevelopment as NDDs are common and the etiology of many is unknown.

## 2. IGF Cellular Signaling

IGF signaling involves two related growth factors, IGF1 and IGF2, that have effects primarily through their receptors IGF1R and IGF2R (Figure 1). IGF1 and IGF2 are small ligands that can bind the IGF receptors with different affinities. IGF1 primarily binds to IGF1R [21,44,45]. IGF2 binds both receptors with high affinity, but IGF1 has slightly higher affinity for IGF1R than IGF2 [21,44]. IGF2 binds both receptors with affinities in the brain still being elucidated for different developmental stages [46]. Less commonly, IGF1 and IGF2 can bind the insulin receptor (InsR), which plays a role in cell growth and metabolism [21,44]. Additionally, there are two isoforms of InsR that can form a hybrid with IGF1R, which IGFs are able to bind [47]. Across tissues, IGF1R is the primary receptor for IGF signaling that promotes proliferation and growth and prevents apoptosis [21,44]. Specifically, IGF1R activation promotes these functions by phosphorylating insulin receptor substrates (IRS) 1-4 and SRC homology collagen (SHC), which then activate the PI3K/AKT and MAPK/ERK pathways [47]. IGF2R, also known as mannose 6-phosphate receptor, signaling is highly distinct in promoting the transport of lysosomal enzymes to lysosomes. Additionally, IGF2R promotes IGF2 degradation that results in downregulation of IGF1R signaling [21,44]. *IGF2* is an imprinted gene in humans and mice, expressed by the paternal copy, while *Igf2r* is only imprinted in mice and is expressed by the maternal copy [48,49]. *IGF2* expression is regulated by *H19*, which is also an imprinted gene expressed from the maternal copy [50]. *H19* encodes a long non-coding RNA that represses *IGF2* expression [51].

IGF1 and IGF2 transport is further regulated by six IGF Binding Proteins (IGFBPs). IGFBPs have both IGF-dependent and IGF-independent functions; for the focus of this review IGF-dependent functions will be primarily discussed. IGF-dependent function of IGFBPs includes two general roles: shuttling IGFs to the receptors and increasing bioavailability or sequestering IGFs away from receptor signaling [52]. IGFs degrade within minutes when unbound, but when bound to IGFBPs, they can remain in circulation for longer periods. This allows IGFs to be shuttled to different organ systems and/or increases bioavailability [52]. Specifically, IGFBP3 and IGFBP5 can increase IGF bioavailability up to 13–15 h as they can form a ternary complex with ALS and IGFs [52,53]. Inhibitory IGFBPs can be cleaved by pregnancy-associated plasma protein A (PAPP-A) and PAPP-A2 to release IGFs for signaling [44,54]. PAPP-A and PAPP-A2 have different substrate preferences. PAPP-A preferentially targets IGFBP2, IGFBP4, and IGFBP5, while PAPP-A2 targets IGFBP3 and IGFBP5 [55]. The IGF signaling pathway, particularly IGF1 and IGF1R, is highly conserved across species, highlighting its critical role in development and homeostasis [56,57,58]. While embryonic and fetal tissues do express *IGF1* and *IGF2,* both IGF1 and IGF2 are produced at high levels in the placenta prior to birth and are necessary for proper development and growth not only for the brain but for the entire body [13,21,44,59,60,61,62,63,64]. It is important to note that tissue-specific expression of IGFs differ across gestation and species. *IGFs* are most highly expressed prenatally and postnatally prior to puberty, after which production of these growth factors decreases throughout life. Postnatal expression of *IGFs* is primarily in the liver, but these factors are also produced at lower levels in other tissues including bone and brain [22,53,65,66]. Additionally, there are many studies that highlight clear sex differences in the IGF signaling pathway, especially sex-specific responses after changes to this pathway, as IGFs interact with sex hormone signaling [13,67,68,69,70,71]. Placental IGF signaling has also been shown to be modulated via sex-specific epigenetic regulation, including microRNAs and methylation, which contributes to sex differences in this pathway [72,73,74]. These studies highlight the importance of considering sex when studying the role of IGF signaling, crucial for understanding the influence of the placenta on neurodevelopment.

## 3. Placental IGF Signaling

While the placenta may be a temporary organ, it is essential for fetal growth and survival. The placenta develops from the trophectoderm alongside the fetus, which develops from the inner cell mass [59]. Both the human and mouse placenta are hemochorial, meaning that fetal tissue comes in direct contact with maternal blood, as the placenta is made up of both maternal and fetal tissue [75]. Maternal blood flow is established at 10–12 weeks gestation in humans, at which point the structure is considered the placenta. Similar establishment of maternal blood flow in the mouse occurs at embryonic day 8.5 (E8.5) [76,77]. The human placenta has highly dynamic vasculature that continues to change throughout gestation, maturing in complexity and function, particularly in the third trimester, to a degree in which the mouse placenta will not [77]. The mouse placenta is also highly dynamic but is considered fully formed and mature by mid-gestation on approximately E12.5 [76]. The human and mouse placenta have a maternal region called the decidua that is responsible for maternal communication and immune protection for the fetus [78]. The mouse placenta is made up of two fetal regions: the junctional zone, which is the primary endocrine region, and the labyrinth zone, which is attached to the umbilical cord and is the primary location for nutrient and hormone production and exchange [76]. The human placenta has one fetal region that has similar functions to the two murine fetal regions [79]. Human and mouse placentas are largely composed of the same cell types [80]. The placenta is made up of placenta-specific cells called trophoblasts. Trophoblast subtypes have specialized functions including hormone production, energy storage and support, and metabolism [80]. Since the placenta is highly vascularized, a large portion of the tissue also comprised endothelial cells that make up the villi [81]. Fetal macrophages known as Hofbauer cells are derived from the yolk sac but reside in the placenta around villi and trophoblasts to aid in placental development and homeostasis [82]. Additionally, decidua cells from the maternal portion of the tissue are not trophoblasts; they are stromal fibroblast-like cells [78]. This region has a high proportion of immune cells, including macrophages, uterine natural killer cells, and T cells [83]. There are also baseline sex differences in placental function in both humans and mice leaving male offspring placentas more susceptible to defects and anomalies [17,84]. In this theoretical framework, male placentas may promote more rapid fetal body growth than females, but this may be at the detriment of other capabilities that may make males more at risk for adverse effects from a multitude of insults [17]. Both human and mouse placentas have high levels of IGF signaling that generally promotes placental growth and function [21].

IGF signaling factors are expressed in different cell types within the placenta. The expression of these factors is largely the same in humans and mice. *IGF1* is primarily expressed from endothelial cells in the fetal region, primarily the labyrinth zone in mice [63,85]. *IGF1* is expressed at a lower level in other placental cells: cytotrophoblasts, syncytiotrophoblasts, as well as decidua cells [63,85]. *IGF2* is more highly expressed in the placenta than *IGF1* [21]. Similar to *IGF1*, *IGF2* is also highly expressed in fetal endothelial cells, but it is also highly expressed in many trophoblasts including cytotrophoblasts (human and mouse), extravillous trophoblasts (human), spongiotrophoblasts (mouse), glycogen cells (mouse), and labyrinth trophoblasts (mouse) [86,87]. The expression of *IGF1* and *IGF2* is dynamic across placental development. *IGF1* generally increases expression across gestation but begins to decrease near parturition [86]. *IGF2* is highly expressed across gestation and peaks immediately before parturition in mice, but its expression across specific cell types varies temporally [86,88]. *IGFBPs* have variable expression across trophoblast and decidua cells, highlighting specific roles for each binding protein [86]. For example, *Igfbp3* and *Igfbp5* are expressed in the mesenchyme of the mouse labyrinth zone [86]. The mesenchyme will become the fetal villi, so expression of these binding proteins in this region aligns with a role of stabilizing IGFs for delivery to the fetus. *IGF1R* and *IGF2R* are expressed primarily in the basal (cytotrophoblasts and syncytiotrophoblasts) and chorionic (fetal endothelial cells) plates of the placenta, which are the communication zones between maternal tissue and placenta as well as placenta and fetus, respectively [86,88,89,90].

The role of IGF signaling in placental development and function has been well studied. IGF1 and IGF2 activate IGF1R signaling, which generally potentiates placental development by promoting trophoblast proliferation, growth, and differentiation, while preventing apoptosis [21]. These functions occur through the downstream activation of PI3K/AKT and MAPK/ERK signaling [21,91]. IGF2 can also activate IGF2R, which can downregulate IGF signaling by initiating degradation of IGFs, as well as increase trophoblast migration and invasion [21,92]. IGF2 and IGF2R signaling promote angiogenesis and proliferation of fetal microvasculature by increasing phosphorylation of ERK1/ERK2 [88]. Currently, IGF1 and IGF1R have not been shown to increase phosphorylation of ERK1/ERK2 within the placenta despite their demonstration of these effects in other tissues. Furthermore, both IGFs promote placental function by upregulating angiogenic factors such as VEGF and PLGF, critical hormones such as progesterone, as well as glucose and amino acid transporters essential for fetal growth [13,21,93,94]. Some studies also demonstrate that IGF2 plays a role in immune balance, particularly between the placenta and uterine environment crucial to maintaining pregnancy and preventing pregnancy loss [95,96]. In sum, IGF signaling regulates and supports proper placental function and growth to support fetal development.

While IGFs support typical placental growth and functioning, they are often involved in contexts of disease and disruption. In general, increased placental IGF1 and IGF2 are associated with increased fetal growth [13,21,97]. An increase in IGF1 impacts labyrinth zone structure. A study that delivered exogenous IGF1 to guinea pig pregnant dams identified a decrease in labyrinth zone size, while another that performed adenoviral-mediated delivery of IGF1 to mouse placentas identified an increase [98,99]. While these studies do not tease out sex-specific findings, another study identified that placental-targeted CRISPR-mediated overexpression of endogenous *Igf1* in mice led to sex-specific changes in labyrinth zone structure [13]. In this study, females displayed an increase in labyrinth zone size while males displayed a decrease [13]. Placental IGF2 increases labyrinth zone area in guinea pigs; additionally, IGF2 levels are positively associated with labyrinth zone size in mice [88,100,101,102]. Additionally, increases in placental IGF signaling can promote vasculature development within the placenta [13,88,102,103]. In contrast, decreased levels of IGF1 and IGF2 reduce fetal weight gain [21,104,105]. Interestingly, decreased placental IGF2 leads to reduced labyrinth zone area and placental mass as well as increased labyrinth zone macrophage population, while lower levels of IGF1, IGF1R, and InsR do not tend to significantly alter placental mass or structure [14,88,104,105,106,107,108,109]. These findings demonstrate overlapping, yet unique roles of IGF1 and IGF2 in placenta structure and function that influence fetal development. Lastly, placental IGF signaling has been shown to respond to environmental cues, such as diet and maternal endocrine challenges but further investigation is needed to tease out the influence of immune challenges. This includes decreases in *Igf2* detected in animal models of undernutrition and low protein diets as well as an increase in placental IGF1 and IGF1R in humans and animal models with maternal diabetes [21,25,110,111,112,113]. Placental IGF signaling is dynamic, shifting across gestation and adapting to environmental influences. This signaling pathway is critical for proper placental development and function, interacts with placental immune processes, and significantly influences fetal growth and subsequent neurodevelopment.

## 4. IGF Signaling in Neurodevelopment

Neurodevelopment is a highly regulated and conserved process that occurs with many similarities across mammals [114]. Changes in neurodevelopment can have substantial effects on function, including cognition, sensory processing, vital automatic processes, and more. Neurodevelopment begins in both humans and mice with the formation of the neural tube, from which the central nervous system develops [59,114,115]. The earliest structure of the brain begins as three primary vesicles that will eventually make up the forebrain (prosencephalon), midbrain (mesencephalon), and hindbrain (rhombencephalon) [115]. Around week 8 of human gestation and E10.5 in mice, this structure differentiates into five secondary vesicles. Two of these vesicles will make up the forebrain (telencephalon and diencephalon), one will become the midbrain (mesencephalon), and two the hindbrain (metencephalon and myelencephalon) [115]. These structures will further differentiate into specialized functional subregions as neurodevelopment continues in utero. The brain is initially composed of neural stem and precursor cells that will differentiate into neurons and glia [114]. Microglia, the immune cells of the brain, enter the embryonic brain from their origins in the yolk sac and begin to divide even before neurogenesis. Neurogenesis occurs almost exclusively in the prenatal period in both humans and mice [114,116]. Human gliogenesis begins in mid-gestation and continues into the early postnatal period. In mice, gliogenesis begins in late gestation and continues further into the postnatal period than in humans [114,116,117]. As cell fate determination proceeds, apoptosis occurs in parallel. Apoptosis is a critical process during neurodevelopment that limits overgrowth of the brain [118]. In both humans and mice, synaptogenesis occurs primarily after birth with synaptic pruning taking place in the juvenile/adolescent period; microglia and immune signaling play a role in the pruning of cells and synapses [114,119]. In mice, myelination begins at birth and stops around postnatal day 60, while in humans, myelination continues over a longer period, beginning perinatally and continuing into early adulthood [114,119,120]. Not unlike the placenta, the human brain develops further during gestation than the mouse. The mouse brain at birth is comparable in many aspects to the second trimester human brain [114,119,121,122]. Another key difference between humans and mice is that the human brain grows significantly larger, allowing for further complexity of the human brain. Both human and mouse brains have sex differences in neurodevelopment modulated most clearly by gonadal hormones, with disruptions to these and other processes such as immune signaling that likely contribute to the risk of adverse neurological and psychiatric conditions [123,124]. The female protectiveness effect is a common phenomenon in which females are less predisposed to these conditions than is seen in humans and in mouse models relevant to these disorders [18,123,125]. While humans and mice have differences in neurodevelopment, there are many conserved developmental processes allowing mice to be a model to understand molecular and cellular mechanisms.

IGF signaling is well established to be critical for neurodevelopment in both humans and mice. Unlike placental IGF signaling studies, which have focused more on IGF2 rather than IGF1, IGF1/IGF1R signaling has been studied more thoroughly in neurodevelopment [22,46,126,127]. This is in part due to the expression patterns of these factors during neurodevelopment. *IGF1R* is ubiquitously expressed in all cells of the developing brain, highlighting its essential role in this tissue [126,127]. Additionally, while in the placenta both IGF1 and IGF2 are shown to promote placental growth and function, primarily IGF1 and IGF1R signaling is essential for proper neurodevelopment. Research in the brain supports that the role of IGF2 and IGF2R signaling is not as critical for neurodevelopment as IGF1R, but instead it is primarily involved in lysosomal trafficking. This is a process that is necessary for brain homeostasis after prenatal and early postnatal development. Additionally, while the roles of IGF1R and IGF2R signaling are well defined within the brain, there has been a lack of research that teases out the extent to which these receptors are activated by either IGF1 or IGF2 at particular time points through development.

*InsR* is also expressed in the developing brain but does not influence neurodevelopment as predominantly as IGF1R signaling [127,128]. IGF1R signaling is essential for proliferation, differentiation, and maturation in the developing brain across species [22,126,127]. *IGF1R* is most highly expressed in neuron-rich areas (gray matter) with minimal expression in white matter; additionally, *IGF1R* is stably expressed in neural stem cells across developmental timepoints [22,127,129]. Interestingly, *IGF1* is also expressed across cell types in the central nervous system, particularly in neurons, and has distinct expression in microglia [22,127,130,131]. *IGF1* expression peaks in the brain early in the postnatal period, and despite *IGF1* being expressed widely across the brain, it is significantly lower expressed than its receptor [22,126,127]. The highest expression of *IGF1R* is in neurons expressing *IGF1* and in cells in close proximity to those producing IGF1, suggesting both an autocrine and paracrine role for IGF1 signaling [22,127,129,132]. In contrast, IGF2 is produced at its highest level within the brain prenatally with decreasing expression postnatally [127,129,133]. *IGF2* is expressed from limited regions of the brain including the choroid plexus, meninges, and blood vessels/endothelial cells [46,127]. Despite restricted expression of *IGF2*, *IGF2R* is more widely expressed in the brain and is enriched in neurons. IGF2R does not have a strong role in neurodevelopment; instead, IGF2R plays a role in brain homeostasis postnatally, specifically lysosomal trafficking [46,134]. Both IGF1 and IGF2 can pass through the blood–brain barrier. While unbound circulating IGFs can pass through the blood–brain barrier, usually circulating IGFs are bound to IGFBPs that increase IGF stability. Currently there have not been studies that track placentally produced IGF factors throughout the fetus, but current models indicate the necessity of placental IGF for neurodevelopment [105,135]. Subsequently, IGF signaling in the brain is stimulated and regulated by external sources of IGFs, such as the placenta [13,22,46,127]. IGFBPs are expressed within the brain, except some studies report no CNS expression of IGFBP1 [127]. IGFBPs are also able to cross the blood–brain barrier and are found in the brain from external sources, including IGFBP1 in CSF [136].

IGF signaling has been shown to significantly influence brain growth. In multiple transgenic mouse models, ubiquitous and conditional *Igf1* and *Igf2* overexpression creates an increase in brain growth and size [13,127,137,138,139,140,141,142]. Different models of *Igf1* overexpression have been shown to increase the volume of several subregions including the cerebral cortex, cerebellum, hippocampus, and striatum [13,127,139,143]. In contrast, ubiquitous and conditional knockouts of *Igf1*, *Igf2*, and *Igf1R* in transgenic mice cause decreases in brain growth and size [105,127,144,145,146,147,148,149,150,151,152]. Knockouts of IGF signaling demonstrate decreased volumes of the same brain regions impacted in *Igf1* overexpression mice [127]. Changes in gross morphology in the brain after changes to IGF signaling highlight the strong influence of this pathway in neurodevelopment. While IGF signaling promotes brain growth, it has not been implicated in early patterning of the brain [144,153]. IGF1 signaling has been well characterized to promote proliferation, differentiation, and anti-apoptotic effects in the developing brain [22,127]. Particularly, IGF1 promotes cell cycle progression by accelerating the G1 phase, subsequently promoting proliferation [13,127,154,155]. Additionally, IGF1 preferentially promotes neuron differentiation and proliferation from neural precursor cells [127,156]. Several studies show an increase in neurons in *Igf1* overexpression models, while *Igf1* and *Igf1R* knockout models demonstrate a decrease in neurons [13,22,105,127,130,139,144,157,158] with evidence in human cells that IGF1 from brain immune cells, microglia, is critical for neuronal proliferation [130], providing an additional pathway by which placental inflammation may influence the brain. Lastly, IGF1 prevents apoptosis by increasing expression of anti-apoptotic factors within neural precursors via IGF1R activation and PI3K/AKT signaling [127,159,160]. IGF1 and IGF1R signaling also promote neurite outgrowth and synaptogenesis [127]. While IGF1 signaling is heavily involved in neurogenesis, IGF1 also promotes the production of oligodendrocytes, astrocytes, and even microglia, as well as their maturation, such as in the creation of myelination [22,127]. Although IGF1 and IGF1R are broadly involved in neurodevelopmental processes, IGF2 and IGF2R have been less implicated in this process. As IGF2 deficiency mouse models display a decrease in brain volume and neural progenitor cell proliferation, there is a clear role for this factor in neurodevelopment [46,104,146]. In vitro, the addition of IGF2 to neural stem cells promotes proliferation and maintenance of the stem cell identity [127,146,161]. These actions are likely mediated through IGF1R or InsR signaling. A global knockout of *Igf2R* is lethal at birth, demonstrating a strong influence in fetal development, though the role of IGF2R in neurodevelopment is not well understood [46,162]. IGF2R has been studied primarily outside of neurodevelopment; in contrast, its function has been studied in the context of aging and memory. IGF2R’s primary signaling pathway within the brain is the initiation of lysosomal trafficking and degradation, a process essential for homeostasis within the brain [46,163].

Due to the essential roles of IGF signaling in neurodevelopment, it is unsurprising that disruptions in this pathway are linked to neurodevelopmental and neurological disorders. Specifically, disruptions in IGF1 levels are associated with neurodevelopmental disorders. While causal impacts of IGF1 pathophysiology have not been determined, evidence suggests that when IGF1 falls out of an expected range, there is an increased risk for neurodevelopmental anomalies. In human studies of juveniles (2 to 15 years old), elevated levels of serum IGF1 have been associated with both ASD and ADHD, while decreased levels of IGF1 in serum and CSF have been associated with ASD [26,27,29,30,31,32,164]. It is important to note that these studies capture different tissues and developmental stages, often with variations in confounding factors such as nutrition or medication unaccounted for. Additionally, mutations in *IGF1* and *IGF1R* in humans are linked with ASD and neurological impairments [26,33,165]. A single nucleotide polymorphism in *IGF1* was associated with decreased IGF1 protein levels in serum in ASD patients, indicating that a deficit of this protein may have occurred due to this mutation and subsequently influenced neurodevelopment [26]. The mutations found in *IGF1R* were found to create early stop codons and occurred in regions responsible for the encoding protein binding areas in the receptor [33,165]. Mutations of this kind are predicted to significantly impair IGF1R signaling as ligands will not be able to properly bind and activate signaling. Aberrant downstream signaling of IGF1R, specifically PI3K/AKT and MAPK/ERK, is often found in non-syndromic and syndromic ASD [166], including Rett syndrome, Phelan–McDermid syndrome, and Fragile X syndrome [167,168,169,170]. While changes to IGF1 and IGF1R are associated with increased risk for neurodevelopmental and neurological disorders, IGF2 and IGF2R are not currently reported to be linked with these conditions. Despite this, IGF2 treatments in mice and IGF1 treatments in humans and mice both in vivo and in vitro have been shown to improve neurobiological and behavioral outcomes [166,171,172,173,174,175,176,177,178]. These findings show the clear importance of IGF signaling for neurodevelopment, as well as their promising role as therapeutics for related conditions. Treatment in the postnatal juvenile period likely impacts some distinct and some overlapping processes from those involving prenatal IGF signaling, making it important to understand placental IGFs’ impact on brain development.

## 5. Association of Placental IGF Signaling and Neurodevelopment

Placental IGF signaling has been implicated in neurodevelopmental disorders from both human and animal studies. These studies have found changes in placental IGF signaling factors in cases of fetal overgrowth, fetal growth restriction (FGR), prematurity, maternal diet differences, and environmental exposures, many of which may have changes induced through inflammatory pathways. While these studies do not isolate the role of placental IGF factors in neurodevelopment, they do associate placental IGF signaling changes with altered brain growth and development outcomes (Table 1). These studies highlight the key role of this signaling pathway in growth and developmental processes, including neurodevelopment.

Several associative studies with human tissue have correlated placental IGF signaling with neurodevelopmental outcomes to garner better insight into its influence in neurodevelopment and contribution to risk of adverse outcomes. A major focus in this area is the influence of placental IGF signaling on neurodevelopment in preterm birth or small for gestational age (SGA) infants. Placental IGF signaling expression is associated with common brain injury, neonatal white matter damage, in extremely low gestational age newborns, defined as less than 28 weeks gestation at birth [179,180]. Both white matter damage and preterm birth are linked to increased ASD-risk and decreased IGF levels, supporting that placental IGF signaling changes may underlie these risks [26,28,34,181,182]. This study from Marable et al. found that decreased growth hormone signaling, including *IGF2* and *IGFBP3,* were associated with neonate white matter damage identified by ultrasound within two months of birth, specifically echolucency [179]. Furthermore, *IGFBP3* was found to be significantly associated with ASD-risk when placental gene expressions associated with white matter damages were compared to the SFARI database of autism relevant genes [179]. Results from male and female neonates were used but no sex differences were described. The authors proposed that changes in the expression of placental IGF genes may influence fetal growth and placental inflammation that may in turn affect neonatal white matter in extremely preterm neonates. A different study by Leviton et al. found that low levels of IGF1 and IGFBP1 from very early postnatal fetal blood samples were associated with FGR in extremely early preterm birth that the authors suggested was likely due to placental insufficiency [183]. The authors suggest that placental insufficiency could lead to insufficient levels of IGF1 being delivered to the fetus [183]. Sex differences were not discussed within this study. Interestingly, infants in the bottom quartile of IGFBP1 levels had an increased risk of developing enlarged lateral ventricles, also known as ventriculomegaly, a condition associated with poor neurodevelopmental outcomes within the first week of life [183,184]. In sum, the study by Marable et al. found a clear link between placental IGF signaling and neurodevelopment after preterm birth in line with the findings from Leviton et al.

Infants that are SGA or experienced FGR are at increased risk for adverse neurodevelopmental outcomes. Children who experienced these conditions are more likely to have cognitive and motor deficits as well as structural differences within the brain [185,186,187,188]. These changes also contribute to an increased risk of NDD diagnosis [185,186,187,188]. Human studies have identified decreased levels of placental IGF1, IGF1R, InsR, IRS2, and PAPP-A in conditions of growth restriction [189,190,191,192,193,194]. Other studies in humans and animal models have found an increase in placental IGFBPs in these conditions, specifically IGFBP1 [189,191,195]. These studies suggest that excess IGFBP levels in the placenta may sequester IGFs away from IGF receptor signaling, subsequently negatively impacting growth. In contrast, conditions such as gestational diabetes and maternal obesity are associated with fetal overgrowth but also linked with increased NDD-risk [25,39,41,42,43]. Human studies have shown increased levels of placental IGF signaling including IGF1, IGF1R, InsR, IRS1, and AKT [25,43,196,197]. Placental IGFBPs, including IGFBP1, are also downregulated in these conditions [43,198]. Mouse studies demonstrate that aberrant placental IGF signaling often results in altered body growth not unlike what is seen in the described human conditions [13,21,44,59,105,106]. These studies show that placental IGF signaling factor levels are often altered in both human and animal growth restriction or overgrowth studies. Changes to placental IGF signaling in these conditions may also contribute to the commensurate increased risk for neurodevelopmental anomalies.

Animal model studies also identified that placental IGF signaling is associated with altered neurodevelopmental outcomes. These studies have often identified placental IGF signaling changes after changes to maternal environment. One study performed by the Bale lab identified sex-specific changes in mouse placental IGF signaling from the offspring of dams that underwent preconception stress [199]. Both maternal preconception stress and stress during pregnancy are associated with altered neurodevelopment in offspring as well as altered placental gene expression [199,200,201,202,203]. In this study, dams were subjected to six weeks of multiple stress methods to elicit chronic stress prior to breeding [199]. Female offspring placentas showed an increase in gene expression associated with regulation of IGF transport and uptake by IGFBPs, specifically *Igfbp1*, *Igbp6*, *Papp-a*, and *Igfals* (encodes ALS) [199]. In contrast, male offspring placentas had a downregulation of this pathway. The authors posited that changes in expression of this pathway would impact the bioavailability of IGFs and subsequently influence offspring neurodevelopment. While not correlated directly to placental IGF changes, transcriptomic analysis of offspring fetal brain also revealed sex-specific alterations in genes involved in neurodevelopment [199] specifically, a decrease in neurotransmitter and ion transport pathways in females and a decrease in neuronal cell fate commitment and proliferation pathways in males [199]. These male fetal brain outcomes after maternal preconception stress are in line with results expected after decreased placental IGF signaling. Furthermore, sex-specific transcriptomic changes to an enriched neuronal population from the hypothalamus across two postnatal timepoints may have been influenced by sex-specific changes in placental expression after maternal preconception stress [199]. Interestingly, an earlier study performed by the same lab found opposite responses in placental *Igfbp1* expression in a mouse model of early maternal prenatal stress [201]. This study instead found an increase in *Igfbp1* and *Glut4* expression in male offspring placentas and a decrease in these factors in females [201]. Furthermore, this study only identified behavioral changes in male offspring, including anhedonia and maladaptive stress responsivity, in addition to neurobiological differences compared to males from naïve dams [201]. Differences in maternal stress timing likely influenced different sex-specific changes to placental IGF signaling in these two studies.

A study investigating the role of maternal immune activation (MIA) in mice found a significant effect on placental IGF signaling. MIA was induced via an injection of poly(I:C) on E12.5 [204]. After MIA, placentas demonstrated a significant increase in the immune factor IL-6. The authors demonstrated that this change influenced placental IGF signaling. When poly(I:C) was administered to pregnant dams, a decrease in placental IGF1 and IGFBP3 gene expression and protein levels was observed, while a complete knockout of IL-6 in combination with poly(I:C) resulted in an increase in IGF1 and IGFBP3 [204]. Additionally, MIA reduced levels of placental GH, another IGF signaling factor. Offspring that experienced MIA showed increased anxiety-like behaviors observed in open field and sensorimotor testing [204]. While unclear if changes in placental IGF signaling are directly related to changes in postnatal behavior, this study demonstrates that placental immune signaling can lead to these outcomes in tandem.

Preeclampsia is a condition seen in pregnancy that is associated with placental dysfunction, especially maladaptive vasculature and inflammation, and is linked to increased neurodevelopmental risks in children [205,206,207,208]. A study from the Stevens lab investigated placental expression, neurobiological, and behavioral changes in offspring from an arginine vasopressin preeclampsia mouse model [205]. While both offspring sexes had decreased embryonic forebrain volumes, adult offspring had sex-specific forebrain changes [205]. Adult females had increased caudate–putamen volume and males had decreased neocortex volume [205]. Furthermore, sex-specific behavioral changes were identified, including impaired procedural learning in females and increased anxiety-like behaviors in males [205]. Transcriptomic analysis revealed different pathways were altered in the embryonic cortex in preeclampsia-exposed males and females that likely influenced the trajectory of adult forebrain morphology and related behavior [205]. Preeclampsia-exposed males but not females had decreased *Igf1* expression [205]. The decrease in placental *Igf1* in males may have contributed to growth changes in the postnatal forebrain. This may have possibly played a part in shaping subsequent behaviors in males. Low protein diet also affects transcriptomics in male and female offspring placentas and fetal brains in rats [209]. Placental *Igf1* and fetal brain *Igf1R* expression were both decreased in response to maternal low protein diet [209]. The authors hypothesize that the reduction in placental *Igf1* may have influenced the downregulation of *Igf1R* in fetal brains and that this may be an adaptive response to changes in maternal environment [209]. Both studies demonstrate possible connections of placental IGF signaling with neurodevelopment.

Associative studies provide critical insight into perinatal conditions that influence placental IGF signaling and, subsequently, brain growth and development. Placental IGF signaling was not directly experimentally manipulated in these studies. Changes observed in neurodevelopment occur due to a combination of multiple changes within the described conditions, including broader placental changes, and are not necessarily due to direct effects of placental IGF in the brain. These studies are especially insightful for understanding placental IGF signaling in the context of conditions of human perinatal adversity which particularly involve immune dysregulation and are known to influence fetal neurodevelopment. Animal studies expand upon these findings, helping to further parse out external factors that may influence placental IGF signaling. Both human and animal studies of this kind have identified placental IGF signaling as a candidate pathway associated with altered neurodevelopment, supporting the need for studies evaluating direct manipulation of this pathway and its effect on fetal development.

**Table 1:** Associations of placental IGF signaling and neurodevelopment.

Study	Species	Condition	Placental IGF Signaling Differences	Neurodevelopmental Outcomes
Marable et al. 2022 [179]	Human	Preterm Birth	Males & Females Decreased GH signaling, including *IGF2* and *IGFBP3*	Males & Females Neonatal white matter damage (echolucency) associated with decreased placental *IGF2* and *IGFBP3*. Placental *IGFBP3* associated with ASD-risk when compared to enriched SFARI genes identified in neonate brain.
Cisse et al. 2024 [199]	Mouse	Maternal Pre-conception Stress	Males Downregulation of genes associated with regulation of IGF transport and uptake by IGFBPs. Females Increased expression of genes associated with regulation of IGF transport and uptake by IGFBPs.	Males Downregulation of neuronal cell fate commitment and proliferation related genes in fetal brains. Sex-specific transcriptional changes in enriched neuronal population in the adolescent and adult hypothalamus. Females Downregulation of neurotransmitter and ion transport-related genes in fetal brains. Sex-specific transcriptional changes in enriched neuronal population in the adolescent and adult hypothalamus.
Mueller and Bale 2008 [201]	Mouse	Maternal Early Prenatal Stress	Males Increased *Igfbp1* and *Glut4* expression. Females Decreased *Igfbp1* and *Glut4* expression.	Males Anhedonia and maladaptive stress responsivity. Neurobiological differences in 5-HT system compared to naïve dam offspring. Females No behavioral differences found.
Hsiao and Patterson 2011 [204]	Mouse	Maternal Immune Activation (Poly(I:C))	Males & Females Decreased IGF1, IGFBP3, and GH levels after administration of poly(I:C) accompanied by increase in IL-6. Increase in IGF1 and IGFBP3 levels after administration of poly(I:C) in IL-6 KO mouse line.	Males & Females Increased anxiety-like behaviors in adult offspring including increased freezing in sensorimotor testing and decreased center time and distance traveled in OFT.
Gumusoglu et al. 2021 [205]	Mouse	Pre-eclampsia (Arginine Vasopressin Mouse Model)	Males Decreased *Igf1* expression.	Males Morphology changes such as decreased embryonic forebrain volumes and decreased adult neocortex volume. Behavior changes include increased anxiety-like behaviors. Sex-specific transcriptional changes in the embryonic cortex.
Fragoso et al. 2021 [209]	Rat	Low Protein Maternal Diet	Males & Females Reduced *Igf1* expression.	Males & Females Reduced *Igf1R* expression in fetal brain.

Underlined sex descriptors indicate which sex(es) the lists below pertain to.

## 6. Experimental Manipulation of Placental IGF Signaling and Neurodevelopmental Outcomes

Animal model studies have been used to directly manipulate placental IGF signaling factors to parse out their influence on the developing brain and subsequent behavior (Table 2). There are few placental-specific or placental-targeted genetic models or techniques, which has been a limitation in this field [210,211]. Several of these studies have employed novel techniques to specifically manipulate placental expression to investigate IGF signaling changes. Genetic techniques were employed in these studies to manipulate placental IGF factor expression in rodents, including Cre–Lox systems, nanoparticle delivery, or CRISPR manipulation [210,212,213,214]. Changes in placental IGF1 and IGF2 production are expected to directly alter neurodevelopment; the trajectory of brain growth may be impacted by abnormal circulating levels of these factors or their binding proteins as they are able to cross the blood–brain barrier and interact with receptors within the brain. While it has not been shown directly that placentally produced IGFs cross the blood–brain barrier, it is believed that IGFs produced in fetal allantoic endothelial cells within the placenta enter fetal circulation that could potentially cross the blood–brain barrier [85,88]. It has been shown that maternally produced IGFs do not cross the placental barrier [215]. It is unclear whether trophoblast-produced IGFs cross into fetal circulation [63]. Direct placental-to-brain delivery has not yet been demonstrated during development. Additionally, changes in placental IGF signaling may indirectly influence neurodevelopment through altered placental function and specifically its capabilities to support fetal development and subsequently neurodevelopment. This may occur as IGF signaling within the placenta has important influences on angiogenic and immune processes. And as described above, placental IGF signaling is in turn influenced by immune signaling [204]. Animal model studies are incredibly important to understand the specific role of placental IGF signaling as well as the implications of dysregulated placental signaling in neurodevelopment.

Mouse studies have shown changes in neurodevelopment and behavior after the knockout or reduction in expression of the two primary growth factors in IGF signaling, *Igf1* and *Igf2*. While IGF1 and IGF2 have some redundancy, they have distinct roles in IGF signaling within the placenta and alter neurodevelopment in unique ways [135]. A study utilized placental-targeted CRISPR manipulation to knock out *Igf1*, creating an insufficiency that is expected to reduce the levels of IGF1 delivered to the developing fetus [105]. Both E18 males and females displayed decreased total forebrain and striatal volume, striatal cell population, and altered transcription within the forebrain. Transcription changes were sex-specific; female forebrains had significant enrichment of DEGs associated with ASD-risk from the SFARI database while males had downregulation of biological processes that are likely to influence neurodevelopment [105]. These changes resulted in sex-specific postnatal forebrain morphology and related behavior outcomes. Males had deficiencies in motor learning, faster reversal learning, and increased stereotyped behaviors accompanied by a decrease in adult striatal neurons [105]. Females had deficiencies in procedural learning, reversal learning, and decreased stereotyped behaviors as well as changes to forebrain white matter development [105]. Interestingly, in both adult males and females there was a significant increase in forebrain white matter astrocytes, cells with substantial immune responsiveness [105]. This is a phenotype seen in other models of preterm birth as well as in human preterm birth studies [180,216,217,218,219,220,221,222,223]. An increase in white matter astrocytes in this model indicates long-term disruption to neurobiology after insufficient placental IGF1 production, showing potential continuous interaction between IGF1 and immune pathways. This study also showed that an existing Cre–Lox system was unable to appropriately knock out placental *Igf1* expression, highlighting the limitations of existing placental-specific genetic manipulation techniques [105]. Another study pharmacologically inhibited IGF1R systemically in early postnatal mice (P1-5) to simulate the loss of placental IGF1 and preterm birth at a timepoint more relevant to third trimester neurodevelopment in humans [121]. While this study did not directly alter placental IGF signaling, it found similar changes to neurodevelopment and behavior as the aforementioned study that created placental *Igf1* insufficiency [121]. These changes included increased stereotyped behaviors and decreased neuronal populations [121]. In sum, placental *Igf1* insufficiency resulted in lifelong neurodevelopmental changes, demonstrating a strong influence of this placentally produced hormone on forebrain development.

Another study highlighted the importance of placental-specific *Igf2* (*Igf2*-P0) expression in embryonic growth and postnatal behavior by comparing *Igf2*-P0 and complete *Igf2* knockout (*Igf2*-total) mouse lines [135]. This study was performed with only male mice. While both *Igf2* knockouts resulted in FGR and reduced birth weights, *Igf2*-P0 knockouts underwent a period of compensatory growth and ultimately reached normal weight by adulthood. In contrast, *Igf2*-total knockouts were smaller across their lifespan when compared to their wildtype counterparts [135]. Only *Igf2*-P0 knockout mice demonstrated increased anxiety-like behaviors in adulthood, demonstrated across multiple behavioral tests including elevated plus maze, open field testing, and acoustic startle response [135]. These behavior changes were accompanied by altered expression of genes associated with anxiety, GABA-Aα3 receptor subunit and 5-HT_2A_ receptor, in the hippocampus [135]. The authors proposed that the differences observed between the complete *Igf2* and placental-specific *Igf2* knockouts can be explained by differences in fetal demand and placental supply. When *Igf2* is completely knocked out, fetal demand and placental supply are reduced proportionally, but when just *Igf2*-P0 is knocked out, early placental capacity is not maintained while fetal demand remains, leading to unmet fetal demand in late gestation [135]. This study distinguishes between placental-specific *Igf2* and *Igf2*-total loss, highlighting a specific and unique role of placentally produced IGF2 in hippocampal function and anxiety-like behaviors.

A placenta-specific *InsR* knockout was performed in mice to tease out its role in fetal neurodevelopment due to known defective placental InsR signaling in pregnancy with metabolic dysfunction often seen in obesity and diabetes [14]. These conditions in pregnancy are associated with adverse fetal neurodevelopmental outcomes [40,224]. This study employed the placenta-specific Cyp-19-cre mouse line bred with an *InsR* flox line to generate homozygous knockout animals for further study [14]. Behavioral testing revealed significant male-biased behavioral changes. Only male *InsR* knockout animals displayed a significant increase in corticosterone after acute restraint stress as well as impaired sensorimotor gating in adulthood [14]. Furthermore, differently expressed genes (DEGs) were found only in male *InsR* knockout prefrontal cortex, a brain region highly linked to NDDs and behaviors assessed in this study [14,225]. Enrichment of nucleotide metabolic processes as well as increased expression of immunomodulatory neuropeptides in prefrontal cortex DEGs along with other findings in this brain region indicated a possible response to mitochondrial dysfunction, oxidative stress, and immune pathways in male knockout animals [14]. Additionally, more extensive alterations were identified in fetal brain and placental gene expression in *InsR* knockout males than females [14]. Lastly, no changes in placental or body mass were observed at any timepoint in this study, suggesting a more specific role of placental InsR signaling for male neurodevelopment.

In addition to placental knockout or insufficiency studies, few studies have been performed to elucidate the role of increased placental IGF signaling on neurodevelopment. Several studies performed in the Jones lab have utilized placental nanoparticle-mediated human IGF1 gene therapy in guinea pigs to increase placental IGF1 production to combat FGR after maternal nutrient restriction [214,226,227]. Studies from this lab identified correction of fetal brain sparing after the use of this technique in a growth restriction model [226,227]. Fetal brain sparing is a phenomenon when increased blood supply is directed to the brain to increase fetal survival in cases of FGR and/or placental insufficiency [228]. Both male and female offspring were assessed in these studies, and findings were present across both sexes [226,227]. Additionally, another study with the same approach from this lab investigated fetal brain expression of two growth factors, *Tgfβ* (also an immune regulator) and *Ctgf*, in mice [229]. These studies support the role of placental IGF1 in proper brain growth and development and IGF1 as a possible treatment that could be leveraged to prevent adverse neurodevelopmental outcomes after FGR.

Another study from the Stevens lab demonstrated adverse neurodevelopmental impacts of excess placental IGF1 through the generation of placental-targeted overexpression of endogenous *Igf1* in wildtype mice via placental-targeted CRISPR manipulation [13,210]. This study aimed to investigate the role of excess placental IGF1 on fetal neurodevelopment, as increased levels of IGF1 are associated with ASD and ADHD [29,30,31,32]. Female placentas responded to the overexpression as expected, upregulating growth promoting signaling as well as altering placental structure in a similar fashion [13]. In contrast, male placentas demonstrated clear compensations against overexpression by downregulating IGF signaling and reducing size of the placental region in which IGF1 is primarily produced [13]. Despite sex-specific alterations in placental function, similar changes to cell cycle regulation were seen in early embryonic striatal development that resulted in an enlarged striatum in male and female late-gestation embryos [13,230]. Juvenile behavioral outcomes were also similar in males and females, demonstrating increased hyperactivity in both [13]. Behavioral changes significantly diverged in males and females in early adulthood. Females had changes to multiple behaviors, including increased anxiety, deficits in working memory, and poorer reversal learning [13]. On the other hand, males only displayed increased stereotyped behaviors, primarily grooming. In line with these findings, no changes to adult male striatal morphology were found, while overexpression females displayed an increase in striatal neuron proportion [13]. The authors posited that sex-specific neurobiological and behavior outcomes were influenced by early divergence in placental function, which may have occurred due to higher placental *Igf1* at baseline in males. Overall, placental *Igf1* overexpression significantly and sex-specifically influences striatal development relevant to NDDs. 

In sum, studies that directly manipulated placental IGF signaling in animal models to tease out these factors’ specific roles in neurodevelopment through possible direct and indirect effects on the developing brain are highly informative. While specificity of manipulation is an important goal of these studies, there are practical limitations to demonstrating the absence of nonplacental, off-target effects; all studies showed some degree of specificity. Therefore, the alterations in brain gene expression, morphology, and behavioral outcomes seen in these studies demonstrate that persistent changes to neurodevelopment occur after abnormal placental IGF signaling. These changes are consistent with both direct and indirect effects of placental IGF signaling changes on fetal neurodevelopment.

**Table 2:** Experimental manipulation of placental IGF signaling and neurodevelopmental outcomes.

Study	Species	IGF Signaling Factor	Manipulation	Neurodevelopmental Outcomes
Carver et al. 2026 [105]	Mouse	*Igf1*	Placental-targeted CRISPR knockout via direct microinjection and electroporation	Males Decreased embryonic forebrain and striatal volume. Reduced striatal cell population. Downregulation of biological processes in the embryonic forebrain. Deficiencies in motor learning, altered reversal behavior, and increased stereotypies. Decreased adult striatal neurons. Increased adult forebrain white matter astrocytes
				Females Decreased embryonic forebrain, striatal, and forebrain white matter volume. Reduced embryonic striatal cell population. Enrichment of ASD-risk genes in embryonic forebrain. Deficiencies in procedural learning and reversal learning. Decreased stereotypies. Increased adult forebrain white matter astrocytes.
Davenport et al. 2025 [226] Wilson et al. 2023 [227] Wilson et al. 2021 [229]	Guinea Pig	*Igf1*	Placental-targeted nanoparticle delivered human *IGF1* with a *Cyp19a1* promoter after maternal nutrient restriction	Males & Females Correction of fetal brain sparing. Changes to growth factor expression (*Tgfβ* and *Ctgf*) in fetal brain.
Carver et al. 2025 [13]	Mouse	*Igf1*	Placental-targeted CRISPR overexpression via direct microinjection and electroporation	Males Changes to cell cycle progression in the embryonic ganglionic eminence. Enlarged embryonic striatum. Increased hyperactivity and stereotypies (primarily grooming).
				Females Changes to cell cycle progression in the embryonic ganglionic eminence. Enlarged embryonic striatum. Increased hyperactivity and anxiety. Deficits in working memory and reversal learning. Increased striatal neuron proportion in adult brain.
Mikaelsson et al. 2013 [135]	Mouse	*Igf2*	Total ablation/ knockout of *Igf2*-P0 (placenta-specific transcript) expression	Males Anxiety-like behavior seen in elevated plus maze, open field testing, and acoustic startle response. Altered hippocampal expression of GABAAα3 receptor subunit and 5-HT2A receptors. Linked to anxiety.
Bronson et al. 2017 [14]	Mouse	*InsR*	*Cyp19*-Cre and *InsR* flox to create placenta-specific knockout	Males Extensive transcriptional alterations in fetal brain. Impaired sensorimotor gating. Increased corticosterone after acute restraint stress. Transcriptional changes in prefrontal cortex, including enrichment in nucleotide metabolic processes.
				Females Changes in fetal brain transcription.

Underlined sex descriptors indicate which sex(es) the lists below pertain to.

## 7. Knowledge Gaps

A focus on the role of placental IGF signaling in neurodevelopment is a fairly new field and requires more investigation to expand our understanding of this critical pathway’s role. Particularly, there is a lack of studies investigating the role of the primary receptors, IGF1R and IGF2R, and the regulatory IGFBPs that may guide organ-specific delivery of IGFs in utero. Additionally, more studies have investigated the knockout or loss of this function, leaving a clear deficit in studies evaluating overexpression or excess production of IGF factors. As IGFs are produced in both trophoblasts and endothelial cells within the placenta, further research is required to parse the role of IGFs in placental function and fetal growth from these distinct sources. Currently, there are no adequate Cre-dependent systems to address these questions in the mouse. Furthermore, methodologies to tag and track IGF and IGFBP production from specific placental sources to determine if and where they travel and act within the fetus would be highly useful. This would be especially interesting in order to characterize the possible direct interactions of placentally produced IGF factors on the developing brain. Lastly, future studies could more precisely identify the developmental trajectory and timing of these placentally produced factors that may give further insight into basic processes of development but also sex differences observed in the reported studies.

Despite the need for further investigation, the current placental manipulation studies clearly demonstrate how placentally expressed IGF factors significantly influence fetal neurodevelopment and how changes to this signaling pathway may be involved in NDD-risk. Clear sex-specific outcomes were found often in the reported studies. Additionally, multiple studies suggest interactions of placental IGF1 with inflammatory pathways matter for brain development, such as changes to both placental IGF signaling and brain development after in utero immune challenge. However, this area requires further investigation; the widespread expression of *Igf1* in placenta suggests that local inflammation may have brain consequences through IGF1 changes but the impacts of placental IGF1 on brain immune cells are another way these systems are closely related. These studies will help inform preventative interventions and treatment for brain outcomes that can be mediated through the placenta, a target much more accessible than the fetal brain.

## 8. Conclusions and Future Directions

The well-established characterization of IGF signaling within the placenta and brain independently highlights the important role of this pathway in both organs. The field of neuroplacentology has connected these two concepts, demonstrating that placental IGF signaling is important for fetal brain growth and supports an important contribution to the trajectory of neurodevelopment, at a basic biological level, and may contribute to risk factors for neurodevelopmental anomalies such as NDDs. Both human and animal models described in this review support that the fetal brain is sensitive and susceptible to changes in placental IGF signaling. This highlights the importance of this signaling pathway in the biological underpinnings of neurodevelopment. This is shown with experimental manipulations of this pathway as well as associative studies with conditions linked to neurodevelopmental anomalies.

The studies described in this review highlight the possibility of placental IGF signaling as a possible mechanism for later NDD-risk that may be a target for therapeutic treatment and intervention. While more research is required to understand the effects of prenatal IGF targeted treatment, postnatal studies show IGF1 and IGF2 treatment positively support and improve neurodevelopment and behavior [166,171,172,173,174,175,176]. Burgeoning research demonstrates that placental IGF1 treatment supports fetal body growth in guinea pigs [97,226,231]. As the placenta is a transient organ that is more accessible than the brain, future studies are essential to determine if, in the long-term, targeted manipulation of placental IGF signaling pathway could play a role in reducing abnormal neurodevelopment in conditions such as gestational diabetes, FGR, placental insufficiency, and more. While promising, more research in this area is required to investigate factors such as how increased and decreased levels of placental IGFs can both alter neurodevelopment and what thresholds of effects may be. More research to define safe thresholds of placental IGF levels across developmental stages would greatly benefit this burgeoning research area. Additionally, changes to immune signaling in these perinatal adversities intersect with placental IGF signaling abnormalities and understanding this connection may inform clinical neurodevelopmental outcomes. Furthermore, promising therapeutics utilizing IGF1 and IGF2 highlight possible avenues of postnatal treatment and intervention for these conditions [121,166,171,172,173,174,175,176]. Future work will help tease out the roles of more factors within the placental IGF signaling pathway and their influence on neurodevelopment. Current work supports these ideas, demonstrating that placental IGF signaling is closely linked with proper neurodevelopment across species.

## Figures and Tables

**Figure 1:**
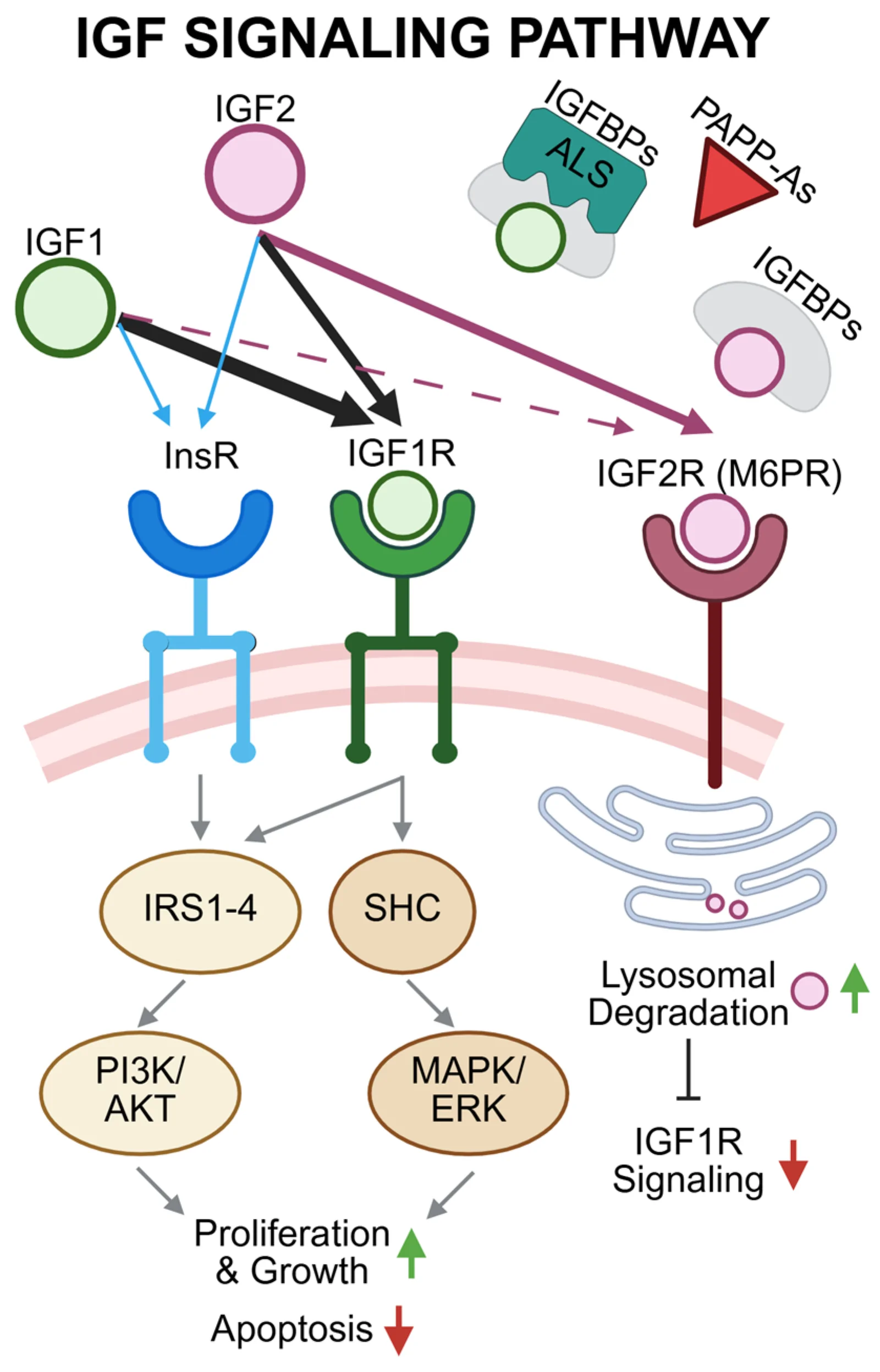
Simplified schematic of IGF signaling. Factors in this signaling pathway are labeled in the figure. Arrows indicate ligand affinity to receptors with a thicker arrow indicating higher affinity, a thin arrow indicating low affinity, and a thin dashed arrow indicating lower affinity. Gray arrows indicate downstream signaling. Green arrows indicate the promotion of biological processes, while red arrows indicate downregulation, and a T-end indicates inhibition. Key signaling outcomes are listed under the receptors. Abbreviations: insulin-like growth factor 1 (IGF1) and 2 (IGF2), IGF1 receptor (IGF1R) and IGF2 receptor (IGF2R), mannose 6-phosphate receptor (M6PR), insulin receptor (InsR), IGF binding proteins (IGFBPs), acid-labile subunit (ALS), pregnancy-associated plasma protein As (PAPP-As), insulin receptor substrates 1-4 (IRS1-4), and SRC homology collagen (SHC).

## Data Availability

No new data were created or analyzed in this study. Data sharing is not applicable to this article.

## Abbreviations

The following abbreviations are used in this manuscript:

ADHD	Attention-deficit/hyperactivity disorder
ASD	Autism spectrum disorder
FGR	Fetal growth restriction
IGF	Insulin-like growth factor
IGFBP	Insulin-like growth factor binding protein
InsR	Insulin receptor
MIA	Maternal immune activation
NDD	Neurodevelopmental disorder
PAPP-A	Pregnancy-associated plasma protein A
SGA	Small for gestational age

## References

[1] Vacher C.M., Bonnin A., Mir I.N., Penn A.A. (2023). Editorial: Advances and perspectives in neuroplacentology. Front. Endocrinol..

[2] Spinillo A., Dominoni M., Mas F.D., Cesari S., Fiandrino G., Gardella B. (2023). Placental fetal vascular malperfusion, neonatal neurologic morbidity, and infant neurodevelopmental outcomes: A systematic review and meta-analysis. Am. J. Obstet. Gynecol..

[3] Nijman M., van der Meeren L.E., Nikkels P.G.J., Stegeman R., Breur J., Jansen N.J.G., Ter Heide H., Steenhuis T.J., de Heus R., Bekker M.N. (2024). Placental Pathology Contributes to Impaired Volumetric Brain Development in Neonates With Congenital Heart Disease. J. Am. Heart Assoc..

[4] Ueda M., Tsuchiya K.J., Yaguchi C., Furuta-Isomura N., Horikoshi Y., Matsumoto M., Suzuki M., Oda T., Kawai K., Itoh T. (2022). Placental pathology predicts infantile neurodevelopment. Sci. Rep..

[5] Lecuyer M., Laquerriere A., Bekri S., Lesueur C., Ramdani Y., Jegou S., Uguen A., Marcorelles P., Marret S., Gonzalez B.J. (2017). PLGF, a placental marker of fetal brain defects after in utero alcohol exposure. Acta Neuropathol. Commun..

[6] Vacher C.M., Lacaille H., O’Reilly J.J., Salzbank J., Bakalar D., Sebaoui S., Liere P., Clarkson-Paredes C., Sasaki T., Sathyanesan A. (2021). Placental endocrine function shapes cerebellar development and social behavior. Nat. Neurosci..

[7] Bakalar D., O’Reilly J.J., Lacaille H., Salzbank J., Ellegood J., Lerch J.P., Sasaki T., Imamura Y., Hashimoto-Torii K., Vacher C.M. (2022). Lack of placental neurosteroid alters cortical development and female somatosensory function. Front. Endocrinol..

[8] Rosenfeld C.S. (2021). The placenta-brain-axis. J. Neurosci. Res..

[9] Bonnin A., Goeden N., Chen K., Wilson M.L., King J., Shih J.C., Blakely R.D., Deneris E.S., Levitt P. (2011). A transient placental source of serotonin for the fetal forebrain. Nature.

[10] Yalcin S.E., Ozler M.R., Yalcin Y., Saglam E., Yilmaz E.S., Nerez N. (2025). Association of Early Pregnancy Inflammatory Indices with Preterm Birth and Perinatal Outcomes in Pregnancies with Pregestational Diabetes. J. Clin. Med..

[11] Lee A.C., Cherkerzian S., Tofail F., Folger L.V., Ahmed S., Rahman S., Chowdhury N.H., Khanam R., Olson I., Oken E. (2024). Perinatal inflammation, fetal growth restriction, and long-term neurodevelopmental impairment in Bangladesh. Pediatr. Res..

[12] Jiang N.M., Cowan M., Moonah S.N., Petri W.A. (2018). The Impact of Systemic Inflammation on Neurodevelopment. Trends Mol. Med..

[13] Carver A.J., Fairbairn F.M., Taylor R.J., Boggarapu S., Kamau N.R., Gajmer A., Stevens H.E. (2025). Placental Igf1 overexpression sex-specifically impacts mouse placenta structure, altering offspring striatal development and behavior. Exp. Neurol..

[14] Bronson S.L., Chan J.C., Bale T.L. (2017). Sex-Specific Neurodevelopmental Programming by Placental Insulin Receptors on Stress Reactivity and Sensorimotor Gating. Biol. Psychiatry.

[15] Salzbank J., Lacaille H., Gaby J., O’Reilly J.J., Kissner M., Vacher C.M., Penn A.A. (2025). Microglia alter sex-specific cerebellar myelination following placental hormone loss. Nat. Commun..

[16] Peric M., Beceheli I., Cicin-Sain L., Desoye G., Stefulj J. (2022). Serotonin system in the human placenta—The knowns and unknowns. Front. Endocrinol..

[17] Eriksson J.G., Kajantie E., Osmond C., Thornburg K., Barker D.J. (2010). Boys live dangerously in the womb. Am. J. Hum. Biol..

[18] Robinson E.B., Lichtenstein P., Anckarsater H., Happe F., Ronald A. (2013). Examining and interpreting the female protective effect against autistic behavior. Proc. Natl. Acad. Sci. USA.

[19] Wigdor E.M., Weiner D.J., Grove J., Fu J.M., Thompson W.K., Carey C.E., Baya N., van der Merwe C., Walters R.K., Satterstrom F.K. (2022). The female protective effect against autism spectrum disorder. Cell Genom..

[20] Taylor M.J., Lichtenstein P., Larsson H., Anckarsater H., Greven C.U., Ronald A. (2016). Is There a Female Protective Effect Against Attention-Deficit/Hyperactivity Disorder? Evidence From Two Representative Twin Samples. J. Am. Acad. Child. Adolesc. Psychiatry.

[21] Sferruzzi-Perri A.N., Sandovici I., Constancia M., Fowden A.L. (2017). Placental phenotype and the insulin-like growth factors: Resource allocation to fetal growth. J. Physiol..

[22] Wrigley S., Arafa D., Tropea D. (2017). Insulin-Like Growth Factor 1: At the Crossroads of Brain Development and Aging. Front. Cell. Neurosci..

[23] Martin-Estal I., de la Garza R.G., Castilla-Cortazar I. (2016). Intrauterine Growth Retardation (IUGR) as a Novel Condition of Insulin-Like Growth Factor-1 (IGF-1) Deficiency. Rev. Physiol. Biochem. Pharmacol..

[24] Hivert M.F., White F., Allard C., James K., Majid S., Aguet F., Ardlie K.G., Florez J.C., Edlow A.G., Bouchard L. (2024). Placental IGFBP1 levels during early pregnancy and the risk of insulin resistance and gestational diabetes. Nat. Med..

[25] Calvo M.J., Parra H., Santeliz R., Bautista J., Luzardo E., Villasmil N., Martinez M.S., Chacin M., Cano C., Checa-Ros A. (2024). The Placental Role in Gestational Diabetes Mellitus: A Molecular Perspective. touchREV. Endocrinol..

[26] Abedini M., Mashayekhi F., Salehi Z. (2022). Analysis of Insulin-like growth factor-1 serum levels and promoter (rs12579108) polymorphism in the children with autism spectrum disorders. J. Clin. Neurosci..

[27] Li Z., Xiao G.Y., He C.Y., Liu X., Fan X., Zhao Y., Wang N.R. (2022). Serum levels of insulin-like growth factor-1 and insulin-like growth factor binding protein-3 in children with autism spectrum disorder. Zhongguo Dang Dai Er Ke Za Zhi.

[28] Riikonen R., Makkonen I., Vanhala R., Turpeinen U., Kuikka J., Kokki H. (2006). Cerebrospinal fluid insulin-like growth factors IGF-1 and IGF-2 in infantile autism. Dev. Med. Child. Neurol..

[29] Allred E.N., Dammann O., Fichorova R.N., Hooper S.R., Hunter S.J., Joseph R.M., Kuban K., Leviton A., O’Shea T.M., Scott M.N. (2017). Systemic Inflammation during the First Postnatal Month and the Risk of Attention Deficit Hyperactivity Disorder Characteristics among 10 year-old Children Born Extremely Preterm. J. Neuroimmune Pharmacol..

[30] Erturk E., Isik U., Sirin F.B. (2023). Analysis of Serum VEGF, IGF-1, and HIF-1alpha Levels in ADHD. J. Atten. Disord..

[31] Mills J.L., Hediger M.L., Molloy C.A., Chrousos G.P., Manning-Courtney P., Yu K.F., Brasington M., England L.J. (2007). Elevated levels of growth-related hormones in autism and autism spectrum disorder. Clin. Endocrinol..

[32] Wang L.J., Huang Y.H., Chou W.J., Lee S.Y., Chang H.Y., Chen C.C., Chao H.R. (2023). Interrelationships among growth hormone, thyroid function, and endocrine-disrupting chemicals on the susceptibility to attention-deficit/hyperactivity disorder. Eur. Child. Adolesc. Psychiatry.

[33] Yang K., Zhang T., Niu R., Zhao L., Cheng Z., Li J., Wang L. (2024). Unveiling the role of IGF1R in autism spectrum disorder: A multi-omics approach to decipher common pathogenic mechanisms in the IGF signaling pathway. Front. Genet..

[34] Crump C., Sundquist J., Sundquist K. (2021). Preterm or Early Term Birth and Risk of Autism. Pediatrics.

[35] Hee Chung E., Chou J., Brown K.A. (2020). Neurodevelopmental outcomes of preterm infants: A recent literature review. Transl. Pediatr..

[36] Hellstrom A., Ley D., Hansen-Pupp I., Hallberg B., Ramenghi L.A., Lofqvist C., Smith L.E., Hard A.L. (2016). Role of Insulinlike Growth Factor 1 in Fetal Development and in the Early Postnatal Life of Premature Infants. Am. J. Perinatol..

[37] Benitez Marin M.J., Blanco Elena J.A., Marin Clavijo J., Jimenez Lopez J., Lubian Lopez D.M., Gonzalez Mesa E. (2022). Neurodevelopment Outcome in Children with Fetal Growth Restriction at Six Years of Age: A Retrospective Cohort Study. Int. J. Environ. Res. Public Health.

[38] Korzeniewski S.J., Allred E.N., Joseph R.M., Heeren T., Kuban K.C.K., O’Shea T.M., Leviton A., Investigators E.S. (2017). Neurodevelopment at Age 10 Years of Children Born < 28 Weeks With Fetal Growth Restriction. Pediatrics.

[39] Dumolt J.H., Powell T.L., Jansson T. (2021). Placental Function and the Development of Fetal Overgrowth and Fetal Growth Restriction. Obstet. Gynecol. Clin. N. Am..

[40] Ye W., Luo C., Zhou J., Liang X., Wen J., Huang J., Zeng Y., Wu Y., Gao Y., Liu Z. (2025). Association between maternal diabetes and neurodevelopmental outcomes in children: A systematic review and meta-analysis of 202 observational studies comprising 56.1 million pregnancies. Lancet Diabetes Endocrinol..

[41] Aviel-Shekler K., Hamshawi Y., Sirhan W., Getselter D., Srikanth K.D., Malka A., Piran R., Elliott E. (2020). Gestational diabetes induces behavioral and brain gene transcription dysregulation in adult offspring. Transl. Psychiatry.

[42] Eleftheriades A., Koulouraki S., Belegrinos A., Eleftheriades M., Pervanidou P. (2025). Maternal Obesity and Neurodevelopment of the Offspring. Nutrients.

[43] Kelly A.C., Powell T.L., Jansson T. (2020). Placental function in maternal obesity. Clin. Sci..

[44] Selenou C., Brioude F., Giabicani E., Sobrier M.L., Netchine I. (2022). IGF2: Development, Genetic and Epigenetic Abnormalities. Cells.

[45] Ewton D.Z., Falen S.L., Florini J.R. (1987). The type II insulin-like growth factor (IGF) receptor has low affinity for IGF-I analogs: Pleiotypic actions of IGFs on myoblasts are apparently mediated by the type I receptor. Endocrinology.

[46] Alberini C.M. (2023). IGF2 in memory, neurodevelopmental disorders, and neurodegenerative diseases. Trends Neurosci..

[47] Hakuno F., Takahashi S.I. (2018). IGF1 receptor signaling pathways. J. Mol. Endocrinol..

[48] Killian J.K., Byrd J.C., Jirtle J.V., Munday B.L., Stoskopf M.K., MacDonald R.G., Jirtle R.L. (2000). M6P/IGF2R imprinting evolution in mammals. Mol. Cell.

[49] Kalscheuer V.M., Mariman E.C., Schepens M.T., Rehder H., Ropers H.H. (1993). The insulin-like growth factor type-2 receptor gene is imprinted in the mouse but not in humans. Nat. Genet..

[50] Nordin M., Bergman D., Halje M., Engstrom W., Ward A. (2014). Epigenetic regulation of the Igf2/H19 gene cluster. Cell Prolif..

[51] Monnier P., Martinet C., Pontis J., Stancheva I., Ait-Si-Ali S., Dandolo L. (2013). H19 lncRNA controls gene expression of the Imprinted Gene Network by recruiting MBD1. Proc. Natl. Acad. Sci. USA.

[52] Bach L.A. (2018). IGF-binding proteins. J. Mol. Endocrinol..

[53] Werner H. (2023). The IGF1 Signaling Pathway: From Basic Concepts to Therapeutic Opportunities. Int. J. Mol. Sci..

[54] Oxvig C. (2015). The role of PAPP-A in the IGF system: Location, location, location. J. Cell Commun. Signal.

[55] Sridar J., Mafi A., Judge R.A., Xu J., Kong K.A., Wang J.C.K., Stoll V.S., Koukos G., Simon R.J., Eaton D. (2023). Cryo-EM structure of human PAPP-A2 and mechanism of substrate recognition. Commun. Chem..

[56] Ali A., Zhang Z.D., Gao T., Aleksic S., Gavathiotis E., Barzilai N., Milman S. (2025). Identification of functional rare coding variants in IGF-1 gene in humans with exceptional longevity. Sci. Rep..

[57] Rotwein P. (2017). Diversification of the insulin-like growth factor 1 gene in mammals. PLoS ONE.

[58] Baral K., Rotwein P. (2019). The insulin-like growth factor 2 gene in mammals: Organizational complexity within a conserved locus. PLoS ONE.

[59] Carver A.J., Dunnwald M., Stevens H.E. (2025). A head start: The relationship of placental factors to craniofacial and brain development. Dev. Dyn..

[60] Steinman G. (2020). The putative etiology and prevention of autism. Prog. Mol. Biol. Transl. Sci..

[61] Liu H.C., He Z.Y., Mele C.A., Veeck L.L., Davis O.K., Rosenwaks Z. (1997). Expression of IGFs and their receptors is a potential marker for embryo quality. Am. J. Reprod. Immunol..

[62] Agrogiannis G.D., Sifakis S., Patsouris E.S., Konstantinidou A.E. (2014). Insulin-like growth factors in embryonic and fetal growth and skeletal development (Review). Mol. Med. Rep..

[63] Hiden U., Glitzner E., Hartmann M., Desoye G. (2009). Insulin and the IGF system in the human placenta of normal and diabetic pregnancies. J. Anat..

[64] Shen S.J., Wang C.Y., Nelson K.K., Jansen M., Ilan J. (1986). Expression of insulin-like growth factor II in human placentas from normal and diabetic pregnancies. Proc. Natl. Acad. Sci. USA.

[65] Netchine I., Azzi S., Le Bouc Y., Savage M.O. (2011). IGF1 molecular anomalies demonstrate its critical role in fetal, postnatal growth and brain development. Best. Pract. Res. Clin. Endocrinol. Metab..

[66] Kawai M., Rosen C.J. (2012). The insulin-like growth factor system in bone: Basic and clinical implications. Endocrinol. Metab. Clin. N. Am..

[67] Fernandez-Arjona M.D.M., Navarro J.A., Lopez-Gambero A.J., de Ceglia M., Rodriguez M., Rubio L., Rodriguez de Fonseca F., Barrios V., Chowen J.A., Argente J. (2024). Sex-based differences in growth-related IGF1 signaling in response to PAPP-A2 deficiency: Comparative effects of rhGH, rhIGF1 and rhPAPP-A2 treatments. Biol. Sex. Differ..

[68] Mauras N., Rogol A.D., Haymond M.W., Veldhuis J.D. (1996). Sex steroids, growth hormone, insulin-like growth factor-1: Neuroendocrine and metabolic regulation in puberty. Horm. Res..

[69] van Bunderen C.C., Deijen J.B., Drent M.L. (2018). Effect of low-normal and high-normal IGF-1 levels on memory and wellbeing during growth hormone replacement therapy: A randomized clinical trial in adult growth hormone deficiency. Health Qual. Life Outcomes.

[70] White V., Jawerbaum A., Mazzucco M.B., Gauster M., Desoye G., Hiden U. (2018). IGF2 stimulates fetal growth in a sex- and organ-dependent manner. Pediatr. Res..

[71] Dai X., He Y., Han X., Sun W., Yu J., Lin Y., Wang Y. (2024). The Regulatory Effect of Insulin-Like Growth Factor-2 on Hypothalamic Gonadotropin-Releasing Hormone Neurons during the Pubertal Period. J. Integr. Neurosci..

[72] Apicella C., Ruano C.S.M., Mehats C., Miralles F., Vaiman D. (2019). The Role of Epigenetics in Placental Development and the Etiology of Preeclampsia. Int. J. Mol. Sci..

[73] Niu Z.R., Han T., Sun X.L., Luan L.X., Gou W.L., Zhu X.M. (2018). MicroRNA-30a-3p is overexpressed in the placentas of patients with preeclampsia and affects trophoblast invasion and apoptosis by its effects on IGF-1. Am. J. Obstet. Gynecol..

[74] Sandovici I., Fernandez-Twinn D.S., Campbell N., Cooper W.N., Sekita Y., Zvetkova I., Ferland-McCollough D., Prosser H.M., Oyama L.M., Pantaleao L.C. (2024). Overexpression of Igf2-derived Mir483 inhibits Igf1 expression and leads to developmental growth restriction and metabolic dysfunction in mice. Cell Rep..

[75] Soncin F., Khater M., To C., Pizzo D., Farah O., Wakeland A., Arul Nambi Rajan K., Nelson K.K., Chang C.W., Moretto-Zita M. (2018). Comparative analysis of mouse and human placentae across gestation reveals species-specific regulators of placental development. Development.

[76] Elmore S.A., Cochran R.Z., Bolon B., Lubeck B., Mahler B., Sabio D., Ward J.M. (2022). Histology Atlas of the Developing Mouse Placenta. Toxicol. Pathol..

[77] Gude N.M., Roberts C.T., Kalionis B., King R.G. (2004). Growth and function of the normal human placenta. Thromb. Res..

[78] Mori M., Bogdan A., Balassa T., Csabai T., Szekeres-Bartho J. (2016). The decidua-the maternal bed embracing the embryo-maintains the pregnancy. Semin. Immunopathol..

[79] Papuchova H., Latos P.A. (2022). Transcription factor networks in trophoblast development. Cell Mol. Life Sci..

[80] Lawless L., Qin Y., Xie L., Zhang K. (2023). Trophoblast Differentiation: Mechanisms and Implications for Pregnancy Complications. Nutrients.

[81] Kalisch-Smith J.I. (2025). Feto-placental blood vessel development. Development.

[82] Yang S.W., Hwang H.S., Kang Y.S. (2025). The role of placenta Hofbauer cells during pregnancy and pregnancy complications. Obstet. Gynecol. Sci..

[83] Menzies F.M. (2025). The Placenta as an Immunological Environment. Br. J. Biomed. Sci..

[84] Braun A.E., Mitchel O.R., Gonzalez T.L., Sun T., Flowers A.E., Pisarska M.D., Winn V.D. (2022). Sex at the interface: The origin and impact of sex differences in the developing human placenta. Biol. Sex. Differ..

[85] Dalcik H., Yardimoglu M., Vural B., Dalcik C., Filiz S., Gonca S., Kokturk S., Ceylan S. (2001). Expression of insulin-like growth factor in the placenta of intrauterine growth-retarded human fetuses. Acta Histochem..

[86] Carter A.M., Nygard K., Mazzuca D.M., Han V.K. (2006). The expression of insulin-like growth factor and insulin-like growth factor binding protein mRNAs in mouse placenta. Placenta.

[87] Aykroyd B.R.L., Tunster S.J., Sferruzzi-Perri A.N. (2020). Igf2 deletion alters mouse placenta endocrine capacity in a sexually dimorphic manner. J. Endocrinol..

[88] Sandovici I., Georgopoulou A., Perez-Garcia V., Hufnagel A., Lopez-Tello J., Lam B.Y.H., Schiefer S.N., Gaudreau C., Santos F., Hoelle K. (2022). The imprinted Igf2-Igf2r axis is critical for matching placental microvasculature expansion to fetal growth. Dev. Cell.

[89] Harris L.K., Crocker I.P., Baker P.N., Aplin J.D., Westwood M. (2011). IGF2 actions on trophoblast in human placenta are regulated by the insulin-like growth factor 2 receptor, which can function as both a signaling and clearance receptor. Biol. Reprod..

[90] Iniguez G., Gonzalez C.A., Argandona F., Kakarieka E., Johnson M.C., Cassorla F. (2010). Expression and protein content of IGF-I and IGF-I receptor in placentas from small, adequate and large for gestational age newborns. Horm. Res. Paediatr..

[91] Forbes K., Westwood M., Baker P.N., Aplin J.D. (2008). Insulin-like growth factor I and II regulate the life cycle of trophoblast in the developing human placenta. Am. J. Physiol. Cell Physiol..

[92] McKinnon T., Chakraborty C., Gleeson L.M., Chidiac P., Lala P.K. (2001). Stimulation of human extravillous trophoblast migration by IGF-II is mediated by IGF type 2 receptor involving inhibitory G protein(s) and phosphorylation of MAPK. J. Clin. Endocrinol. Metab..

[93] de Vrijer B., Davidsen M.L., Wilkening R.B., Anthony R.V., Regnault T.R. (2006). Altered placental and fetal expression of IGFs and IGF-binding proteins associated with intrauterine growth restriction in fetal sheep during early and mid-pregnancy. Pediatr. Res..

[94] Rak-Mardyla A., Gregoraszczuk E.L. (2010). Effect of pituitary growth hormone and insulin-like growth factor type-I on proliferation, apoptosis and hormone secretion of the placental cell line JEG-3. Gynecol. Endocrinol..

[95] Agaoglu O.K., Agaoglu A.R., Guzeloglu A., Aslan S., Kurar E., Kayis S.A., Schafer-Somi S. (2016). Gene expression profiles of some cytokines, growth factors, receptors, and enzymes (GM-CSF, IFNgamma, MMP-2, IGF-II, EGF, TGF-beta, IGF-IIR) during pregnancy in the cat uterus. Theriogenology.

[96] Chen D., Li Q., Yang H., Shi J., Chen P., Wang T., Zhou L., Dai W., Zheng L., Li M. (2026). PTN/IGF-2 signaling modulates endometrial decidualization and immune cell trafficking to facilitate pregnancy maintenance. Front. Immunol..

[97] Jones H.N., Crombleholme T., Habli M. (2013). Adenoviral-mediated placental gene transfer of IGF-1 corrects placental insufficiency via enhanced placental glucose transport mechanisms. PLoS ONE.

[98] Standen P., Sferruzzi-Perri A.N., Taylor R., Heinemann G., Zhang J.V., Highet A.R., Pringle K.G., Owens J.A., Kumarasamy V., Lumbers E.R. (2015). Maternal insulin-like growth factor 1 and 2 differentially affect the renin-angiotensin system during pregnancy in the guinea pig. Growth Horm. IGF Res..

[99] Katz A.B., Keswani S.G., Habli M., Lim F.Y., Zoltick P.W., Midrio P., Kozin E.D., Herlyn M., Crombleholme T.M. (2009). Placental gene transfer: Transgene screening in mice for trophic effects on the placenta. Am. J. Obstet. Gynecol..

[100] Sferruzzi-Perri A.N., Owens J.A., Pringle K.G., Robinson J.S., Roberts C.T. (2006). Maternal insulin-like growth factors-I and -II act via different pathways to promote fetal growth. Endocrinology.

[101] Sferruzzi-Perri A.N., Owens J.A., Standen P., Taylor R.L., Robinson J.S., Roberts C.T. (2007). Early pregnancy maternal endocrine insulin-like growth factor I programs the placenta for increased functional capacity throughout gestation. Endocrinology.

[102] Sferruzzi-Perri A.N., Owens J.A., Standen P., Roberts C.T. (2008). Maternal insulin-like growth factor-II promotes placental functional development via the type 2 IGF receptor in the guinea pig. Placenta.

[103] Davenport B.N., Wilson R.L., Williams A.A., Gray J.A., Stanley E.L., Jones H.N. (2025). Aberrant placental structure is corrected with repeated nanoparticle-mediated IGF1 treatments in a Guinea pig model of fetal growth restriction. Transl. Res. Anat..

[104] Baker J., Liu J.P., Robertson E.J., Efstratiadis A. (1993). Role of insulin-like growth factors in embryonic and postnatal growth. Cell.

[105] Carver A.J., Fairbairn F.M., Taylor R.J., Hing B.W.Q., Gajmer A., Fair R.T., Stevens H.E. (2026). Placental Insulin-like Growth Factor 1 Insufficiency Drives Neurodevelopmental Disorder-Relevant Behavioral Changes with Sex-Specific Vulnerabilities. bioRxiv.

[106] Constancia M., Hemberger M., Hughes J., Dean W., Ferguson-Smith A., Fundele R., Stewart F., Kelsey G., Fowden A., Sibley C. (2002). Placental-specific IGF-II is a major modulator of placental and fetal growth. Nature.

[107] Sibley C.P., Coan P.M., Ferguson-Smith A.C., Dean W., Hughes J., Smith P., Reik W., Burton G.J., Fowden A.L., Constancia M. (2004). Placental-specific insulin-like growth factor 2 (Igf2) regulates the diffusional exchange characteristics of the mouse placenta. Proc. Natl. Acad. Sci. USA.

[108] DeChiara T.M., Efstratiadis A., Robertson E.J. (1990). A growth-deficiency phenotype in heterozygous mice carrying an insulin-like growth factor II gene disrupted by targeting. Nature.

[109] Louvi A., Accili D., Efstratiadis A. (1997). Growth-promoting interaction of IGF-II with the insulin receptor during mouse embryonic development. Dev. Biol..

[110] McMullen S., Osgerby J.C., Milne J.S., Wallace J.M., Wathes D.C. (2005). The effects of acute nutrient restriction in the mid-gestational ewe on maternal and fetal nutrient status, the expression of placental growth factors and fetal growth. Placenta.

[111] Nusken K.D., Schneider H., Plank C., Trollmann R., Nusken E., Rascher W., Dotsch J. (2011). Fetal programming of gene expression in growth-restricted rats depends on the cause of low birth weight. Endocrinology.

[112] White V., Jawerbaum A., Mazzucco M.B., Gauster M., Desoye G., Hiden U. (2015). Diabetes-associated changes in the fetal insulin/insulin-like growth factor system are organ specific in rats. Pediatr. Res..

[113] Hauguel-de Mouzon S., Louizeau M., Girard J. (1992). Functional alterations of type I insulin-like growth factor receptor in placenta of diabetic rats. Biochem. J..

[114] Semple B.D., Blomgren K., Gimlin K., Ferriero D.M., Noble-Haeusslein L.J. (2013). Brain development in rodents and humans: Identifying benchmarks of maturation and vulnerability to injury across species. Prog. Neurobiol..

[115] Richtsmeier J.T., Flaherty K. (2013). Hand in glove: Brain and skull in development and dysmorphogenesis. Acta Neuropathol..

[116] Qian X., Shen Q., Goderie S.K., He W., Capela A., Davis A.A., Temple S. (2000). Timing of CNS cell generation: A programmed sequence of neuron and glial cell production from isolated murine cortical stem cells. Neuron.

[117] Zhang X., Mennicke C.V., Xiao G., Beattie R., Haider M.A., Hippenmeyer S., Ghashghaei H.T. (2020). Clonal Analysis of Gliogenesis in the Cerebral Cortex Reveals Stochastic Expansion of Glia and Cell Autonomous Responses to Egfr Dosage. Cells.

[118] Yamaguchi Y., Miura M. (2015). Programmed cell death in neurodevelopment. Dev. Cell.

[119] Zeiss C.J. (2021). Comparative Milestones in Rodent and Human Postnatal Central Nervous System Development. Toxicol. Pathol..

[120] Snaidero N., Simons M. (2014). Myelination at a glance. J. Cell Sci..

[121] Potenzieri A., Uccella S., Preiti D., Pisoni M., Rosati S., Lavarello C., Bartolucci M., Debellis D., Catalano F., Petretto A. (2024). Early IGF-1 receptor inhibition in mice mimics preterm human brain disorders and reveals a therapeutic target. Sci. Adv..

[122] Gumusoglu S.B., Stevens H.E. (2019). Maternal Inflammation and Neurodevelopmental Programming: A Review of Preclinical Outcomes and Implications for Translational Psychiatry. Biol. Psychiatry.

[123] Breach M.R., Lenz K.M. (2023). Sex Differences in Neurodevelopmental Disorders: A Key Role for the Immune System. Curr. Top. Behav. Neurosci..

[124] Bordt E.A., Moya H.A., Jo Y.C., Ravichandran C.T., Bankowski I.M., Ceasrine A.M., McDougle C.J., Carlezon W.A., Bilbo S.D. (2024). Gonadal hormones impart male-biased behavioral vulnerabilities to immune activation via microglial mitochondrial function. Brain Behav. Immun..

[125] Jeon S.J., Gonzales E.L., Mabunga D.F.N., Valencia S.T., Kim D.G., Kim Y., Adil K.J.L., Shin D., Park D., Shin C.Y. (2018). Sex-specific Behavioral Features of Rodent Models of Autism Spectrum Disorder. Exp. Neurobiol..

[126] Dyer A.H., Vahdatpour C., Sanfeliu A., Tropea D. (2016). The role of Insulin-Like Growth Factor 1 (IGF-1) in brain development, maturation and neuroplasticity. Neuroscience.

[127] O’Kusky J., Ye P. (2012). Neurodevelopmental effects of insulin-like growth factor signaling. Front. Neuroendocrinol..

[128] Girbau M., Bassas L., Alemany J., de Pablo F. (1989). In situ autoradiography and ligand-dependent tyrosine kinase activity reveal insulin receptors and insulin-like growth factor I receptors in prepancreatic chicken embryos. Proc. Natl. Acad. Sci. USA.

[129] Bondy C., Werner H., Roberts C.T., LeRoith D. (1992). Cellular pattern of type-I insulin-like growth factor receptor gene expression during maturation of the rat brain: Comparison with insulin-like growth factors I and II. Neuroscience.

[130] Yu D., Jain S., Wangzhou A., Zhu B., Shao W., Coley-O’Rourke E.J., De Florencio S., Kim J., Choi J.J., Paredes M.F. (2025). Microglia regulate GABAergic neurogenesis in prenatal human brain through IGF1. Nature.

[131] Rusin D., Vahl Becirovic L., Lyszczarz G., Krueger M., Benmamar-Badel A., Vad Mathiesen C., Sigurethardottir Schioth E., Lykke Lambertsen K., Wlodarczyk A. (2024). Microglia-Derived Insulin-like Growth Factor 1 Is Critical for Neurodevelopment. Cells.

[132] Cheng C.M., Reinhardt R.R., Lee W.H., Joncas G., Patel S.C., Bondy C.A. (2000). Insulin-like growth factor 1 regulates developing brain glucose metabolism. Proc. Natl. Acad. Sci. USA.

[133] Ayer-le Lievre C., Stahlbom P.A., Sara V.R. (1991). Expression of IGF-I and -II mRNA in the brain and craniofacial region of the rat fetus. Development.

[134] Wang Y., MacDonald R.G., Thinakaran G., Kar S. (2017). Insulin-Like Growth Factor-II/Cation-Independent Mannose 6-Phosphate Receptor in Neurodegenerative Diseases. Mol. Neurobiol..

[135] Mikaelsson M.A., Constancia M., Dent C.L., Wilkinson L.S., Humby T. (2013). Placental programming of anxiety in adulthood revealed by Igf2-null models. Nat. Commun..

[136] Bunn R.C., King W.D., Winkler M.K., Fowlkes J.L. (2005). Early developmental changes in IGF-I, IGF-II, IGF binding protein-1, and IGF binding protein-3 concentration in the cerebrospinal fluid of children. Pediatr. Res..

[137] Popken G.J., Hodge R.D., Ye P., Zhang J., Ng W., O’Kusky J.R., D’Ercole A.J. (2004). In vivo effects of insulin-like growth factor-I (IGF-I) on prenatal and early postnatal development of the central nervous system. Eur. J. Neurosci..

[138] Carson M.J., Behringer R.R., Brinster R.L., McMorris F.A. (1993). Insulin-like growth factor I increases brain growth and central nervous system myelination in transgenic mice. Neuron.

[139] Gutierrez-Ospina G., Calikoglu A.S., Ye P., D’Ercole A.J. (1996). In vivo effects of insulin-like growth factor-I on the development of sensory pathways: Analysis of the primary somatic sensory cortex (S1) of transgenic mice. Endocrinology.

[140] Dentremont K.D., Ye P., D’Ercole A.J., O’Kusky J.R. (1999). Increased insulin-like growth factor-I (IGF-I) expression during early postnatal development differentially increases neuron number and growth in medullary nuclei of the mouse. Brain Res. Dev. Brain Res..

[141] Ye P., Popken G.J., Kemper A., McCarthy K., Popko B., D’Ercole A.J. (2004). Astrocyte-specific overexpression of insulin-like growth factor-I promotes brain overgrowth and glial fibrillary acidic protein expression. J. Neurosci. Res..

[142] Eggenschwiler J., Ludwig T., Fisher P., Leighton P.A., Tilghman S.M., Efstratiadis A. (1997). Mouse mutant embryos overexpressing IGF-II exhibit phenotypic features of the Beckwith-Wiedemann and Simpson-Golabi-Behmel syndromes. Genes. Dev..

[143] Ye P., Xing Y., Dai Z., D’Ercole A.J. (1996). In vivo actions of insulin-like growth factor-I (IGF-I) on cerebellum development in transgenic mice: Evidence that IGF-I increases proliferation of granule cell progenitors. Brain Res. Dev. Brain Res..

[144] Beck K.D., Powell-Braxton L., Widmer H.R., Valverde J., Hefti F. (1995). Igf1 gene disruption results in reduced brain size, CNS hypomyelination, and loss of hippocampal granule and striatal parvalbumin-containing neurons. Neuron.

[145] Ye P., Li L., Richards R.G., DiAugustine R.P., D’Ercole A.J. (2002). Myelination is altered in insulin-like growth factor-I null mutant mice. J. Neurosci..

[146] Lehtinen M.K., Zappaterra M.W., Chen X., Yang Y.J., Hill A.D., Lun M., Maynard T., Gonzalez D., Kim S., Ye P. (2011). The cerebrospinal fluid provides a proliferative niche for neural progenitor cells. Neuron.

[147] Liu J.P., Baker J., Perkins A.S., Robertson E.J., Efstratiadis A. (1993). Mice carrying null mutations of the genes encoding insulin-like growth factor I (Igf-1) and type 1 IGF receptor (Igf1r). Cell.

[148] Holzenberger M., Leneuve P., Hamard G., Ducos B., Perin L., Binoux M., Le Bouc Y. (2000). A targeted partial invalidation of the insulin-like growth factor I receptor gene in mice causes a postnatal growth deficit. Endocrinology.

[149] Holzenberger M., Hamard G., Zaoui R., Leneuve P., Ducos B., Beccavin C., Perin L., Le Bouc Y. (2001). Experimental IGF-I receptor deficiency generates a sexually dimorphic pattern of organ-specific growth deficits in mice, affecting fat tissue in particular. Endocrinology.

[150] Liu W., Ye P., O’Kusky J.R., D’Ercole A.J. (2009). Type 1 insulin-like growth factor receptor signaling is essential for the development of the hippocampal formation and dentate gyrus. J. Neurosci. Res..

[151] Kappeler L., De Magalhaes Filho C., Dupont J., Leneuve P., Cervera P., Perin L., Loudes C., Blaise A., Klein R., Epelbaum J. (2008). Brain IGF-1 receptors control mammalian growth and lifespan through a neuroendocrine mechanism. PLoS Biol..

[152] Zeger M., Popken G., Zhang J., Xuan S., Lu Q.R., Schwab M.H., Nave K.A., Rowitch D., D’Ercole A.J., Ye P. (2007). Insulin-like growth factor type 1 receptor signaling in the cells of oligodendrocyte lineage is required for normal in vivo oligodendrocyte development and myelination. Glia.

[153] Schlueter P.J., Peng G., Westerfield M., Duan C. (2007). Insulin-like growth factor signaling regulates zebrafish embryonic growth and development by promoting cell survival and cell cycle progression. Cell Death Differ..

[154] Mairet-Coello G., Tury A., DiCicco-Bloom E. (2009). Insulin-like growth factor-1 promotes G_1_/S cell cycle progression through bidirectional regulation of cyclins and cyclin-dependent kinase inhibitors via the phosphatidylinositol 3-kinase/Akt pathway in developing rat cerebral cortex. J. Neurosci..

[155] Hodge R.D., D’Ercole A.J., O’Kusky J.R. (2004). Insulin-like growth factor-I accelerates the cell cycle by decreasing G_1_ phase length and increases cell cycle reentry in the embryonic cerebral cortex. J. Neurosci..

[156] Nieto-Estévez V., Defterali Ç., Vicario-Abejón C. (2016). IGF-I: A Key Growth Factor that Regulates Neurogenesis and Synaptogenesis from Embryonic to Adult Stages of the Brain. Front. Neurosci..

[157] Arsenijevic Y., Weiss S. (1998). Insulin-like growth factor-I is a differentiation factor for postmitotic CNS stem cell-derived neuronal precursors: Distinct actions from those of brain-derived neurotrophic factor. J. Neurosci..

[158] Ye P., Carson J., D’Ercole A.J. (1995). In vivo actions of insulin-like growth factor-I (IGF-I) on brain myelination: Studies of IGF-I and IGF binding protein-1 (IGFBP-1) transgenic mice. J. Neurosci..

[159] Chrysis D., Calikoglu A.S., Ye P., D’Ercole A.J. (2001). Insulin-like growth factor-I overexpression attenuates cerebellar apoptosis by altering the expression of Bcl family proteins in a developmentally specific manner. J. Neurosci..

[160] Baker N.L., Carlo Russo V., Bernard O., D’Ercole A.J., Werther G.A. (1999). Interactions between bcl-2 and the IGF system control apoptosis in the developing mouse brain. Brain Res. Dev. Brain Res..

[161] Ziegler A.N., Schneider J.S., Qin M., Tyler W.A., Pintar J.E., Fraidenraich D., Wood T.L., Levison S.W. (2012). IGF-II promotes stemness of neural restricted precursors. Stem Cells.

[162] Lau M.M., Stewart C.E., Liu Z., Bhatt H., Rotwein P., Stewart C.L. (1994). Loss of the imprinted IGF2/cation-independent mannose 6-phosphate receptor results in fetal overgrowth and perinatal lethality. Genes. Dev..

[163] Yu X.W., Pandey K., Katzman A.C., Alberini C.M. (2020). A role for CIM6P/IGF2 receptor in memory consolidation and enhancement. eLife.

[164] Riikonen R. (2006). Insulin-like growth factor delivery across the blood-brain barrier. Potential use of IGF-1 as a drug in child neurology. Chemotherapy.

[165] Kerem L., Rips J., Zaretsky A., Grundwag I., Cohen E., Yanovsky-Dagan S., Harel T. (2025). Intronic IGF1R variant causing aberrant splicing, short stature, and neurological impairments. J. Mol. Endocrinol..

[166] Rethelyi J.M., Vincze K., Schall D., Glennon J., Berkel S. (2023). The role of insulin/IGF1 signalling in neurodevelopmental and neuropsychiatric disorders—Evidence from human neuronal cell models. Neurosci. Biobehav. Rev..

[167] Li Y., Wang H., Muffat J., Cheng A.W., Orlando D.A., Loven J., Kwok S.M., Feldman D.A., Bateup H.S., Gao Q. (2013). Global transcriptional and translational repression in human-embryonic-stem-cell-derived Rett syndrome neurons. Cell Stem Cell.

[168] Bidinosti M., Botta P., Kruttner S., Proenca C.C., Stoehr N., Bernhard M., Fruh I., Mueller M., Bonenfant D., Voshol H. (2016). CLK2 inhibition ameliorates autistic features associated with SHANK3 deficiency. Science.

[169] Hoeffer C.A., Sanchez E., Hagerman R.J., Mu Y., Nguyen D.V., Wong H., Whelan A.M., Zukin R.S., Klann E., Tassone F. (2012). Altered mTOR signaling and enhanced CYFIP2 expression levels in subjects with fragile X syndrome. Genes. Brain Behav..

[170] Kumar V., Zhang M.X., Swank M.W., Kunz J., Wu G.Y. (2005). Regulation of dendritic morphogenesis by Ras-PI3K-Akt-mTOR and Ras-MAPK signaling pathways. J. Neurosci..

[171] Kolevzon A., Bush L., Wang A.T., Halpern D., Frank Y., Grodberg D., Rapaport R., Tavassoli T., Chaplin W., Soorya L. (2014). A pilot controlled trial of insulin-like growth factor-1 in children with Phelan-McDermid syndrome. Mol. Autism.

[172] Castro J., Garcia R.I., Kwok S., Banerjee A., Petravicz J., Woodson J., Mellios N., Tropea D., Sur M. (2014). Functional recovery with recombinant human IGF1 treatment in a mouse model of Rett Syndrome. Proc. Natl. Acad. Sci. USA.

[173] Costales J., Kolevzon A. (2016). The therapeutic potential of insulin-like growth factor-1 in central nervous system disorders. Neurosci. Biobehav. Rev..

[174] Deacon R.M., Glass L., Snape M., Hurley M.J., Altimiras F.J., Biekofsky R.R., Cogram P. (2015). NNZ-2566, a novel analog of (1-3) IGF-1, as a potential therapeutic agent for fragile X syndrome. Neuromolecular Med..

[175] Steinmetz A.B., Stern S.A., Kohtz A.S., Descalzi G., Alberini C.M. (2018). Insulin-Like Growth Factor II Targets the mTOR Pathway to Reverse Autism-Like Phenotypes in Mice. J. Neurosci..

[176] Cruz E., Descalzi G., Steinmetz A., Scharfman H.E., Katzman A., Alberini C.M. (2021). CIM6P/IGF-2 Receptor Ligands Reverse Deficits in Angelman Syndrome Model Mice. Autism Res..

[177] de Souza J.S., Carromeu C., Torres L.B., Araujo B.H., Cugola F.R., Maciel R.M., Muotri A.R., Giannocco G. (2017). IGF1 neuronal response in the absence of MECP2 is dependent on TRalpha 3. Hum. Mol. Genet..

[178] Williams E.C., Zhong X., Mohamed A., Li R., Liu Y., Dong Q., Ananiev G.E., Mok J.C., Lin B.R., Lu J. (2014). Mutant astrocytes differentiated from Rett syndrome patients-specific iPSCs have adverse effects on wild-type neurons. Hum. Mol. Genet..

[179] Marable C.A., Roell K., Kuban K., O’Shea T.M., Fry R.C. (2022). Placental transcriptional signatures associated with cerebral white matter damage in the neonate. Front. Neurosci..

[180] Back S.A. (2017). White matter injury in the preterm infant: Pathology and mechanisms. Acta Neuropathol..

[181] Campbell H., Check J., Kuban K.C.K., Leviton A., Joseph R.M., Frazier J.A., Douglass L.M., Roell K., Allred E.N., Fordham L.A. (2021). Neonatal Cranial Ultrasound Findings among Infants Born Extremely Preterm: Associations with Neurodevelopmental Outcomes at 10 Years of Age. J. Pediatr..

[182] Hansen-Pupp I., Hovel H., Lofqvist C., Hellstrom-Westas L., Fellman V., Huppi P.S., Hellstrom A., Ley D. (2013). Circulatory insulin-like growth factor-I and brain volumes in relation to neurodevelopmental outcome in very preterm infants. Pediatr. Res..

[183] Leviton A., Allred E.N., Fichorova R.N., VanderVeen D.K., O’Shea T.M., Kuban K., Dammann O., Investigators E.S. (2019). Early Postnatal IGF-1 and IGFBP-1 Blood Levels in Extremely Preterm Infants: Relationships with Indicators of Placental Insufficiency and with Systemic Inflammation. Am. J. Perinatol..

[184] Pappas A., Adams-Chapman I., Shankaran S., McDonald S.A., Stoll B.J., Laptook A.R., Carlo W.A., Van Meurs K.P., Hintz S.R., Carlson M.D. (2018). Neurodevelopmental and Behavioral Outcomes in Extremely Premature Neonates With Ventriculomegaly in the Absence of Periventricular-Intraventricular Hemorrhage. JAMA Pediatr..

[185] Rao A., Suvarna R., Shenoy Belle V., Udupa E.G.P., Lewis L.E.S. (2025). Neurodevelopmental outcomes in Intrauterine Growth-Restricted children—An umbrella review. J. Matern. Fetal Neonatal Med..

[186] Kong L., Huang S., Zhang J., Ji L., Duan S., Xu X., Tian S., Shi F., Wu F., Shen D. (2025). Effect of fetal growth restriction on the development of regional brain volume and neurodevelopmental outcomes: Evidence from magnetic resonance imaging based on a fully automated segmentation method. Magn. Reson. Imaging.

[187] Ferguson K.K., Sammallahti S., Rosen E., van den Dries M., Pronk A., Spaan S., Guxens M., Tiemeier H., Gaillard R., Jaddoe V.W.V. (2021). Fetal Growth Trajectories Among Small for Gestational Age Babies and Child Neurodevelopment. Epidemiology.

[188] Vollmer B., Edmonds C.J. (2019). School Age Neurological and Cognitive Outcomes of Fetal Growth Retardation or Small for Gestational Age Birth Weight. Front. Endocrinol..

[189] Nawathe A.R., Christian M., Kim S.H., Johnson M., Savvidou M.D., Terzidou V. (2016). Insulin-like growth factor axis in pregnancies affected by fetal growth disorders. Clin. Epigenet..

[190] Sandoval C., Wu G., Smith S.B., Dunlap K.A., Satterfield M.C. (2020). Maternal Nutrient Restriction and Skeletal Muscle Development: Consequences for Postnatal Health. Adv. Exp. Med. Biol..

[191] Akram S.K., Carlsson-Skwirut C., Bhutta Z.A., Soder O. (2011). Placental IGF-I, IGFBP-1, zinc, and iron, and maternal and infant anthropometry at birth. Acta Paediatr..

[192] Laviola L., Perrini S., Belsanti G., Natalicchio A., Montrone C., Leonardini A., Vimercati A., Scioscia M., Selvaggi L., Giorgino R. (2005). Intrauterine growth restriction in humans is associated with abnormalities in placental insulin-like growth factor signaling. Endocrinology.

[193] Morris R.K., Bilagi A., Devani P., Kilby M.D. (2017). Association of serum PAPP-A levels in first trimester with small for gestational age and adverse pregnancy outcomes: Systematic review and meta-analysis. Prenat. Diagn..

[194] Potau N., Riudor E., Ballabriga A. (1981). Insulin receptors in human placenta in relation to fetal weight and gestational age. Pediatr. Res..

[195] Crossey P.A., Pillai C.C., Miell J.P. (2002). Altered placental development and intrauterine growth restriction in IGF binding protein-1 transgenic mice. J. Clin. Investig..

[196] Jansson N., Rosario F.J., Gaccioli F., Lager S., Jones H.N., Roos S., Jansson T., Powell T.L. (2013). Activation of placental mTOR signaling and amino acid transporters in obese women giving birth to large babies. J. Clin. Endocrinol. Metab..

[197] Tumminia A., Scalisi N.M., Milluzzo A., Ettore G., Vigneri R., Sciacca L. (2021). Maternal Diabetes Impairs Insulin and IGF-1 Receptor Expression and Signaling in Human Placenta. Front. Endocrinol..

[198] Altmae S., Segura M.T., Esteban F.J., Bartel S., Brandi P., Irmler M., Beckers J., Demmelmair H., Lopez-Sabater C., Koletzko B. (2017). Maternal Pre-Pregnancy Obesity Is Associated with Altered Placental Transcriptome. PLoS ONE.

[199] Cisse Y.M., Montgomery K.R., Zierden H.C., Hill E.M., Kane P.J., Huang W., Kane M.A., Bale T.L. (2024). Maternal preconception stress produces sex-specific effects at the maternal:fetal interface to impact offspring development and phenotypic outcomesdagger. Biol. Reprod..

[200] Mina T.H., Raikkonen K., Riley S.C., Norman J.E., Reynolds R.M. (2015). Maternal distress associates with placental genes regulating fetal glucocorticoid exposure and IGF2: Role of obesity and sex. Psychoneuroendocrinology.

[201] Mueller B.R., Bale T.L. (2008). Sex-specific programming of offspring emotionality after stress early in pregnancy. J. Neurosci..

[202] Maurer S.V., Hing B.W.Q., Lussier S., Radhakrishna S., Davis J.L.B., Abbott P.W., Michaelson J.J., Stevens H.E. (2025). Prenatal stress alters mouse offspring dorsal striatal development and placental function in sex-specific ways. J. Psychiatr. Res..

[203] Kundu S., Maurer S.V., Stevens H.E. (2021). Future Horizons for Neurodevelopmental Disorders: Placental Mechanisms. Front. Pediatr..

[204] Hsiao E.Y., Patterson P.H. (2011). Activation of the maternal immune system induces endocrine changes in the placenta via IL-6. Brain Behav. Immun..

[205] Gumusoglu S.B., Chilukuri A.S.S., Hing B.W.Q., Scroggins S.M., Kundu S., Sandgren J.A., Santillan M.K., Santillan D.A., Grobe J.L., Stevens H.E. (2021). Altered offspring neurodevelopment in an arginine vasopressin preeclampsia model. Transl. Psychiatry.

[206] Gumusoglu S.B., Chilukuri A.S.S., Santillan D.A., Santillan M.K., Stevens H.E. (2020). Neurodevelopmental Outcomes of Prenatal Preeclampsia Exposure. Trends Neurosci..

[207] Walker C.K., Krakowiak P., Baker A., Hansen R.L., Ozonoff S., Hertz-Picciotto I. (2015). Preeclampsia, placental insufficiency, and autism spectrum disorder or developmental delay. JAMA Pediatr..

[208] Opichka M.A., Rappelt M.W., Gutterman D.D., Grobe J.L., McIntosh J.J. (2021). Vascular Dysfunction in Preeclampsia. Cells.

[209] Fragoso J., Carvalho Jurema Santos G., da Silva H.T., Loizon E., de Oliveira Nogueira Souza V., Vidal H., Guedes R.C.A., Costa-Silva J.H., da Silva Aragao R., Pirola L. (2021). Effects of maternal low-protein diet and spontaneous physical activity on the transcription of neurotrophic factors in the placenta and the brains of mothers and offspring rats. J. Dev. Orig. Health Dis..

[210] Carver A.J., Taylor R.J., Stevens H.E. (2023). Mouse In Vivo Placental Targeted CRISPR Manipulation. J. Vis. Exp..

[211] Renaud S.J., Karim Rumi M.A., Soares M.J. (2011). Review: Genetic manipulation of the rodent placenta. Placenta.

[212] Zhou C.C., Chang J., Mi T., Abbasi S., Gu D., Huang L., Zhang W., Kellems R.E., Schwartz R.J., Xia Y. (2012). Targeted expression of Cre recombinase provokes placental-specific DNA recombination in transgenic mice. PLoS ONE.

[213] Wenzel P.L., Leone G. (2007). Expression of Cre recombinase in early diploid trophoblast cells of the mouse placenta. Genesis.

[214] Wilson R.L., Lampe K., Gupta M.K., Duvall C.L., Jones H.N. (2022). Nanoparticle-mediated transgene expression of insulin-like growth factor 1 in the growth restricted guinea pig placenta increases placenta nutrient transporter expression and fetal glucose concentrations. Mol. Reprod. Dev..

[215] Sferruzzi-Perri A.N., Owens J.A., Standen P., Taylor R.L., Heinemann G.K., Robinson J.S., Roberts C.T. (2007). Early treatment of the pregnant guinea pig with IGFs promotes placental transport and nutrient partitioning near term. Am. J. Physiol. Endocrinol. Metab..

[216] Mallard E.C., Rees S., Stringer M., Cock M.L., Harding R. (1998). Effects of chronic placental insufficiency on brain development in fetal sheep. Pediatr. Res..

[217] Duncan J.R., Cock M.L., Loeliger M., Louey S., Harding R., Rees S.M. (2004). Effects of exposure to chronic placental insufficiency on the postnatal brain and retina in sheep. J. Neuropathol. Exp. Neurol..

[218] Wixey J.A., Lee K.M., Miller S.M., Goasdoue K., Colditz P.B., Tracey Bjorkman S., Chand K.K. (2019). Neuropathology in intrauterine growth restricted newborn piglets is associated with glial activation and proinflammatory status in the brain. J. Neuroinflamm..

[219] Roessmann U., Gambetti P. (1986). Pathological reaction of astrocytes in perinatal brain injury. Immunohistochemical study. Acta Neuropathol..

[220] Mallard C., Davidson J.O., Tan S., Green C.R., Bennet L., Robertson N.J., Gunn A.J. (2014). Astrocytes and microglia in acute cerebral injury underlying cerebral palsy associated with preterm birth. Pediatr. Res..

[221] Li L., Cui X., Zhu B., Zhou L., Guo Y., Liu T., Shi Y. (2025). Role of astrocytes in the pathogenesis of perinatal brain injury. Mol. Med..

[222] Chen Q., Zhang K., Wang M., Gao R., Wang Q., Xiao M., Chen C. (2022). A translational mouse model for investigation of the mechanism of preterm diffuse white matter injury. Transl. Pediatr..

[223] Van Steenwinckel J., Bokobza C., Laforge M., Shearer I.K., Miron V.E., Rua R., Matta S.M., Hill-Yardin E.L., Fleiss B., Gressens P. (2024). Key roles of glial cells in the encephalopathy of prematurity. Glia.

[224] Rivera H.M., Christiansen K.J., Sullivan E.L. (2015). The role of maternal obesity in the risk of neuropsychiatric disorders. Front. Neurosci..

[225] Schubert D., Martens G.J., Kolk S.M. (2015). Molecular underpinnings of prefrontal cortex development in rodents provide insights into the etiology of neurodevelopmental disorders. Mol. Psychiatry.

[226] Davenport B.N., Wilson R.L., Williams A.A., Jones H.N. (2025). Placental nanoparticle-mediated IGF1 gene therapy corrects fetal growth restriction in a guinea pig model. Gene Ther..

[227] Wilson R.L., Stephens K.K., Jones H.N. (2023). Placental nanoparticle gene therapy normalizes gene expression changes in the fetal liver associated with fetal growth restriction in a fetal sex-specific manner. J. Dev. Orig. Health Dis..

[228] Roza S.J., Steegers E.A., Verburg B.O., Jaddoe V.W., Moll H.A., Hofman A., Verhulst F.C., Tiemeier H. (2008). What is spared by fetal brain-sparing? Fetal circulatory redistribution and behavioral problems in the general population. Am. J. Epidemiol..

[229] Wilson R.L., Stephens K.K., Lampe K., Jones H.N. (2021). Sexual dimorphisms in brain gene expression in the growth-restricted guinea pig can be modulated with intra-placental therapy. Pediatr. Res..

[230] Evans M.M., Kim J., Abel T., Nickl-Jockschat T., Stevens H.E. (2024). Developmental Disruptions of the Dorsal Striatum in Autism Spectrum Disorder. Biol. Psychiatry.

[231] Wilson R.L., Schmidt J.K., Davenport B.N., Ren E., Keding L.T., Shaw S.A., Schotzko M.L., Antony K.M., Simmons H.A., Golos T.G. (2024). Placental gene therapy in nonhuman primates: A pilot study of maternal, placental, and fetal response to non-viral, polymeric nanoparticle delivery of IGF1. Mol. Hum. Reprod..

